# SARS-CoV-2-specific nasal IgA wanes 9 months after hospitalisation with COVID-19 and is not induced by subsequent vaccination

**DOI:** 10.1016/j.ebiom.2022.104402

**Published:** 2022-12-19

**Authors:** Felicity Liew, Shubha Talwar, Andy Cross, Brian J. Willett, Sam Scott, Nicola Logan, Matthew K. Siggins, Dawid Swieboda, Jasmin K. Sidhu, Claudia Efstathiou, Shona C. Moore, Chris Davis, Noura Mohamed, Jose Nunag, Clara King, A.A. Roger Thompson, Sarah L. Rowland-Jones, Annemarie B. Docherty, James D. Chalmers, Ling-Pei Ho, Alexander Horsley, Betty Raman, Krisnah Poinasamy, Michael Marks, Onn Min Kon, Luke Howard, Daniel G. Wootton, Susanna Dunachie, Jennifer K. Quint, Rachael A. Evans, Louise V. Wain, Sara Fontanella, Thushan I. de Silva, Antonia Ho, Ewen Harrison, J. Kenneth Baillie, Malcolm G. Semple, Christopher Brightling, Ryan S. Thwaites, Lance Turtle, Peter J.M. Openshaw, ISARIC4C Investigators, J. Kenneth Baillie, Peter J.M. Openshaw, Malcolm G. Semple, Beatrice Alex, Petros Andrikopoulos, Benjamin Bach, Wendy S. Barclay, Debby Bogaert, Meera Chand, Kanta Chechi, Graham S. Cooke, Ana da Silva Filipe, Thushan de Silva, Annemarie B. Docherty, Gonçalo dos Santos Correia, Marc-Emmanuel Dumas, Jake Dunning, Tom Fletcher, Christopher A. Green, William Greenhalf, Julian Griffin, Rishi K. Gupta, Ewen M. Harrison, Antonia Y.W. Ho, Karl Holden, Peter W. Horby, Samreen Ijaz, Say Khoo, Paul Klenerman, Andrew Law, Matthew Lewis, Sonia Liggi, Wei Shen Lim, Lynn Maslen, Alexander J. Mentzer, Laura Merson, Alison M Meynert, Shona C. Moore, Mahdad Noursadeghi, Michael Olanipekun, Anthonia Osagie, Massimo Palmarini, Carlo Palmieri, William A. Paxton, Georgios Pollakis, Nicholas Price, Andrew Rambaut, David L Robertson, Clark D. Russell, Vanessa Sancho-Shimizu, Caroline Sands, Janet T. Scott, Louise Sigfrid, Tom Solomon, Shiranee Sriskandan, David Stuart, Charlotte Summers, Olivia V. Swann, Zoltan Takats, Panteleimon Takis, Richard S. Tedder, A.A. Roger Thompson, Emma C. Thomson, Ryan S. Thwaites, Lance C.W. Turtle, Maria Zambon, Thomas M. Drake, Cameron J. Fairfield, Stephen R. Knight, Kenneth A. Mclean, Derek Murphy, Lisa Norman, Riinu Pius, Catherine A. Shaw, Marie Connor, Jo Dalton, Carrol Gamble, Michelle Girvan, Sophie Halpin, Janet Harrison, Clare Jackson, James Lee, Laura Marsh, Daniel Plotkin, Stephanie Roberts, Egle Saviciute, Sara Clohisey, Ross Hendry, Susan Knight, Eva Lahnsteiner, Andrew Law, Gary Leeming, Lucy Norris, James Scott-Brown, Sarah Tait, Murray Wham, Richard Clark, Audrey Coutts, Lorna Donelly, Angie Fawkes, Tammy Gilchrist, Katarzyna Hafezi, Louise MacGillivray, Alan Maclean, Sarah McCafferty, Kirstie Morrice, Lee Murphy, Nicola Wrobel, Gail Carson, Kayode Adeniji, Daniel Agranoff, Ken Agwuh, Dhiraj Ail, Erin L. Aldera, Ana Alegria, Sam Allen, Brian Angus, Abdul Ashish, Dougal Atkinson, Shahedal Bari, Gavin Barlow, Stella Barnass, Nicholas Barrett, Christopher Bassford, Sneha Basude, David Baxter, Michael Beadsworth, Jolanta Bernatoniene, John Berridge, Colin Berry, Nicola Best, Pieter Bothma, Robin Brittain-Long, Naomi Bulteel, Tom Burden, Andrew Burtenshaw, Vikki Caruth, David Chadwick, David Chadwick, Duncan Chambler, Nigel Chee, Jenny Child, Srikanth Chukkambotla, Tom Clark, Paul Collini, Catherine Cosgrove, Jason Cupitt, Maria-Teresa Cutino-Moguel, Paul Dark, Chris Dawson, Samir Dervisevic, Phil Donnison, Sam Douthwaite, Andrew Drummond, Ingrid DuRand, Ahilanadan Dushianthan, Tristan Dyer, Cariad Evans, Chi Eziefula, Chrisopher Fegan, Adam Finn, Duncan Fullerton, Sanjeev Garg, Sanjeev Garg, Atul Garg, Effrossyni Gkrania-Klotsas, Jo Godden, Arthur Goldsmith, Clive Graham, Tassos Grammatikopoulos, Elaine Hardy, Stuart Hartshorn, Daniel Harvey, Peter Havalda, Daniel B. Hawcutt, Maria Hobrok, Luke Hodgson, Anil Hormis, Joanne Howard, Michael Jacobs, Susan Jain, Paul Jennings, Agilan Kaliappan, Vidya Kasipandian, Stephen Kegg, Michael Kelsey, Jason Kendall, Caroline Kerrison, Ian Kerslake, Oliver Koch, Gouri Koduri, George Koshy, Shondipon Laha, Steven Laird, Susan Larkin, Tamas Leiner, Patrick Lillie, James Limb, Vanessa Linnett, Jeff Little, Mark Lyttle, Michael MacMahon, Emily MacNaughton, Ravish Mankregod, Huw Masson, Elijah Matovu, Katherine McCullough, Ruth McEwen, Manjula Meda, Gary Mills, Jane Minton, Kavya Mohandas, Quen Mok, James Moon, Elinoor Moore, Patrick Morgan, Craig Morris, Katherine Mortimore, Samuel Moses, Mbiye Mpenge, Rohinton Mulla, Michael Murphy, Thapas Nagarajan, Megan Nagel, Mark Nelson, Lillian Norris, Matthew K. O'Shea, Marlies Ostermann, Igor Otahal, Mark Pais, Carlo Palmieri, Selva Panchatsharam, Danai Papakonstantinou, Padmasayee Papineni, Hassan Paraiso, Brij Patel, Natalie Pattison, Justin Pepperell, Mark Peters, Mandeep Phull, Stefania Pintus, Tim Planche, Frank Post, David Price, Rachel Prout, Nikolas Rae, Henrik Reschreiter, Tim Reynolds, Neil Richardson, Mark Roberts, Devender Roberts, Alistair Rose, Guy Rousseau, Bobby Ruge, Brendan Ryan, Taranprit Saluja, Sarah Sarah, Matthias Schmid, Aarti Shah, Manu Shankar-Hari, Prad Shanmuga, Anil Sharma, Anna Shawcross, Jagtur Singh Pooni, Jeremy Sizer, Richard Smith, Catherine Snelson, Nick Spittle, Nikki Staines, Tom Stambach, Richard Stewart, Pradeep Subudhi, Tamas Szakmany, Kate Tatham, Jo Thomas, Chris Thompson, Robert Thompson, Ascanio Tridente, Darell Tupper-Carey, Mary Twagira, Nick Vallotton, Rama Vancheeswaran, Rachel Vincent, Lisa Vincent-Smith, Shico Visuvanathan, Alan Vuylsteke, Sam Waddy, Rachel Wake, Andrew Walden, Ingeborg Welters, Tony Whitehouse, Paul Whittaker, Ashley Whittington, Meme Wijesinghe, Martin Williams, Lawrence Wilson, Stephen Winchester, Martin Wiselka, Adam Wolverson, Daniel G Wootton, Andrew Workman, Bryan Yates, Peter Young, Sarah E. McDonald, Victoria Shaw, Katie A. Ahmed, Jane A. Armstrong, Milton Ashworth, Innocent G. Asiimwe, Siddharth Bakshi, Samantha L Barlow, Laura Booth, Benjamin Brennan, Katie Bullock, Nicola Carlucci, Emily Cass, Benjamin W.A. Catterall, Jordan J. Clark, Emily A. Clarke, Sarah Cole, Louise Cooper, Helen Cox, Christopher Davis, Oslem Dincarslan, Alejandra Doce Carracedo, Chris Dunn, Philip Dyer, Angela Elliott, Anthony Evans, Lorna Finch, Lewis W.S. Fisher, Lisa Flaherty, Terry Foster, Isabel Garcia-Dorival, Philip Gunning, Catherine Hartley, Anthony Holmes, Rebecca L. Jensen, Christopher B. Jones, Trevor R. Jones, Shadia Khandaker, Katharine King, Robyn T. Kiy, Chrysa Koukorava, Annette Lake, Suzannah Lant, Diane Latawiec, Lara Lavelle-Langham, Daniella Lefteri, Lauren Lett, Lucia A Livoti, Maria Mancini, Hannah Massey, Nicole Maziere, Sarah McDonald, Laurence McEvoy, John McLauchlan, Soeren Metelmann, Nahida S. Miah, Joanna Middleton, Joyce Mitchell, Shona C. Moore, Ellen G Murphy, Rebekah Penrice-Randal, Jack Pilgrim, Tessa Prince, Will Reynolds, P. Matthew Ridley, Debby Sales, Victoria E. Shaw, Rebecca K. Shears, Benjamin Small, Krishanthi S. Subramaniam, Agnieska Szemiel, Aislynn Taggart, Jolanta Tanianis-Hughes, Jordan Thomas, Erwan Trochu, Libby van Tonder, Eve Wilcock, J. Eunice Zhang, Seán Keating, Cara Donegan, Rebecca G. Spencer, Chloe Donohue, Fiona Griffiths, Hayley Hardwick, Wilna Oosthuyzen, PHOSP-COVID collaborative group, K. Abel, H. Adamali, D. Adeloye, O. Adeyemi, R. Adrego, L.A. Aguilar Jimenez, S. Ahmad, N. Ahmad Haider, R. Ahmed, N. Ahwireng, M. Ainsworth, B. Al-Sheklly, A. Alamoudi, M. Ali, M. Aljaroof, A.M. All, L. Allan, R.J. Allen, L. Allerton, L. Allsop, P. Almeida, D. Altmann, M. Alvarez Corral, S. Amoils, D. Anderson, C. Antoniades, G. Arbane, A. Arias, C. Armour, L. Armstrong, N. Armstrong, D. Arnold, H. Arnold, A. Ashish, A. Ashworth, M. Ashworth, S. Aslani, H. Assefa-Kebede, C. Atkin, P. Atkin, R. Aul, H. Aung, L. Austin, C. Avram, A. Ayoub, M. Babores, R. Baggott, J. Bagshaw, D. Baguley, L. Bailey, J.K. Baillie, S. Bain, M. Bakali, M. Bakau, E. Baldry, D. Baldwin, M. Baldwin, C. Ballard, A. Banerjee, B. Bang, R.E. Barker, L. Barman, S. Barratt, F. Barrett, D. Basire, N. Basu, M. Bates, A. Bates, R. Batterham, H. Baxendale, H. Bayes, M. Beadsworth, P. Beckett, M. Beggs, M. Begum, P. Beirne, D. Bell, R. Bell, K. Bennett, E. Beranova, A. Bermperi, A. Berridge, C. Berry, S. Betts, E. Bevan, K. Bhui, M. Bingham, K. Birchall, L. Bishop, K. Bisnauthsing, J. Blaikely, A. Bloss, A. Bolger, C.E. Bolton, J. Bonnington, A. Botkai, C. Bourne, M. Bourne, K. Bramham, L. Brear, G. Breen, J. Breeze, A. Briggs, E. Bright, C.E. Brightling, S. Brill, K. Brindle, L. Broad, A. Broadley, C. Brookes, M. Broome, A. Brown, A. Brown, J. Brown, J. Brown, J.S. Brown, M. Brown, M. Brown, V. Brown, T. Brugha, N. Brunskill, M. Buch, P. Buckley, A. Bularga, E. Bullmore, L. Burden, T. Burdett, D. Burn, G. Burns, A. Burns, J. Busby, R. Butcher, A. Butt, S. Byrne, P. Cairns, P.C. Calder, E. Calvelo, H. Carborn, B. Card, C. Carr, L. Carr, G. Carson, P. Carter, A. Casey, M. Cassar, J. Cavanagh, M. Chablani, T. Chalder, J.D. Chalmers, R.C. Chambers, F. Chan, K.M. Channon, K. Chapman, A. Charalambou, N. Chaudhuri, A. Checkley, J. Chen, Y. Cheng, L. Chetham, C. Childs, E.R. Chilvers, H. Chinoy, A. Chiribiri, K. Chong-James, G. Choudhury, N. Choudhury, P. Chowienczyk, C. Christie, M. Chrystal, D. Clark, C. Clark, J. Clarke, S. Clohisey, G. Coakley, Z. Coburn, S. Coetzee, J. Cole, C. Coleman, F. Conneh, D. Connell, B. Connolly, L. Connor, A. Cook, B. Cooper, J. Cooper, S. Cooper, D. Copeland, T. Cosier, M. Coulding, C. Coupland, E. Cox, T. Craig, P. Crisp, D. Cristiano, M.G. Crooks, A. Cross, I. Cruz, P. Cullinan, D. Cuthbertson, L. Daines, M. Dalton, P. Daly, A. Daniels, P. Dark, J. Dasgin, A. David, C. David, E. Davies, F. Davies, G. Davies, G.A. Davies, K. Davies, M.J. Davies, J. Dawson, E. Daynes, A. De Soyza, B. Deakin, A. Deans, C. Deas, J. Deery, S. Defres, A. Dell, K. Dempsey, E. Denneny, J. Dennis, A. Dewar, R. Dharmagunawardena, N. Diar-Bakerly, C. Dickens, A. Dipper, S. Diver, S.N. Diwanji, M. Dixon, R. Djukanovic, H. Dobson, S.L. Dobson, A.B. Docherty, A. Donaldson, T. Dong, N. Dormand, A. Dougherty, R. Dowling, S. Drain, K. Draxlbauer, K. Drury, P. Dulawan, A. Dunleavy, S. Dunn, C. Dupont, J. Earley, N. Easom, C. Echevarria, S. Edwards, C. Edwardson, H. El-Taweel, A. Elliott, K. Elliott, Y. Ellis, A. Elmer, O. Elneima, D. Evans, H. Evans, J. Evans, R. Evans, R.A. Evans, R.I. Evans, T. Evans, C. Evenden, L. Evison, L. Fabbri, S. Fairbairn, A. Fairman, K. Fallon, D. Faluyi, C. Favager, T. Fayzan, J. Featherstone, T. Felton, J. Finch, S. Finney, J. Finnigan, L. Finnigan, H. Fisher, S. Fletcher, R. Flockton, M. Flynn, H. Foot, D. Foote, A. Ford, D. Forton, E. Fraile, C. Francis, R. Francis, S. Francis, A. Frankel, E. Fraser, R. Free, N. French, X. Fu, J. Fuld, J. Furniss, L. Garner, N. Gautam, J.R. Geddes, J. George, P. George, M. Gibbons, M. Gill, L. Gilmour, F. Gleeson, J. Glossop, S. Glover, N. Goodman, C. Goodwin, B. Gooptu, H. Gordon, T. Gorsuch, M. Greatorex, P.L. Greenhaff, W. Greenhalf, A. Greenhalgh, N.J. Greening, J. Greenwood, H. Gregory, R. Gregory, D. Grieve, D. Griffin, L. Griffiths, A-M. Guerdette, B. Guillen Guio, M. Gummadi, A. Gupta, S. Gurram, E. Guthrie, Z. Guy, H. H Henson, K. Hadley, A. Haggar, K. Hainey, B. Hairsine, P. Haldar, I. Hall, L. Hall, M. Halling-Brown, R. Hamil, A. Hancock, K. Hancock, N.A. Hanley, S. Haq, H.E. Hardwick, E. Hardy, T. Hardy, B. Hargadon, K. Harrington, E. Harris, V.C. Harris, E.M. Harrison, P. Harrison, N. Hart, A. Harvey, M. Harvey, M. Harvie, L. Haslam, M. Havinden-Williams, J. Hawkes, N. Hawkings, J. Haworth, A. Hayday, M. Haynes, J. Hazeldine, T. Hazelton, L.G. Heaney, C. Heeley, J.L. Heeney, M. Heightman, S. Heller, M. Henderson, L. Hesselden, M. Hewitt, V. Highett, T. Hillman, T. Hiwot, L.P. Ho, A. Hoare, M. Hoare, J. Hockridge, P. Hogarth, A. Holbourn, S. Holden, L. Holdsworth, D. Holgate, M. Holland, L. Holloway, K. Holmes, M. Holmes, B. Holroyd-Hind, L. Holt, A. Hormis, A. Horsley, A. Hosseini, M. Hotopf, L. Houchen-Wolloff, K. Howard, L.S. Howard, A. Howell, E. Hufton, A.D. Hughes, J. Hughes, R. Hughes, A. Humphries, N. Huneke, E. Hurditch, J. Hurst, M. Husain, T. Hussell, J. Hutchinson, W. Ibrahim, F. Ilyas, J. Ingham, L. Ingram, D. Ionita, K. Isaacs, K. Ismail, T. Jackson, J. Jacob, W.Y. James, W. Jang, C. Jarman, I. Jarrold, H. Jarvis, R. Jastrub, B. Jayaraman, R.G. Jenkins, P. Jezzard, K. Jiwa, C. Johnson, S. Johnson, D. Johnston, C.J. Jolley, D. Jones, G. Jones, H. Jones, H. Jones, I. Jones, L. Jones, M.G. Jones, S. Jones, S. Jose, T. Kabir, G. Kaltsakas, V. Kamwa, N. Kanellakis, s. Kaprowska, Z. Kausar, N. Keenan, S. Kelly, G. Kemp, S. Kerr, H. Kerslake, A.L. Key, F. Khan, K. Khunti, S. Kilroy, B. King, C. King, L. Kingham, J. Kirk, P. Kitterick, P. Klenerman, L. Knibbs, S. Knight, A. Knighton, O. Kon, S. Kon, S.S. Kon, S. Koprowska, A. Korszun, I. Koychev, C. Kurasz, P. Kurupati, C. Laing, H. Lamlum, G. Landers, C. Langenberg, D. Lasserson, L. Lavelle-Langham, A. Lawrie, C. Lawson, C. Lawson, A. Layton, A. Lea, O.C. Leavy, D. Lee, J-H. Lee, E. Lee, K. Leitch, R. Lenagh, D. Lewis, J. Lewis, K.E. Lewis, V. Lewis, N. Lewis-Burke, X. Li, T. Light, L. Lightstone, W. Lilaonitkul, L. Lim, S. Linford, A. Lingford-Hughes, M. Lipman, K. Liyanage, A. Lloyd, S. Logan, D. Lomas, N.I. Lone, R. Loosley, J.M. Lord, H. Lota, W. Lovegrove, A. Lucey, E. Lukaschuk, A. Lye, C. Lynch, S. MacDonald, G. MacGowan, I. Macharia, J. Mackie, L. Macliver, S. Madathil, G. Madzamba, N. Magee, M.M. Magtoto, N. Mairs, N. Majeed, E. Major, F. Malein, M. Malim, G. Mallison, W.D.-C. Man, S. Mandal, K. Mangion, C. Manisty, R. Manley, K. March, S. Marciniak, P. Marino, M. Mariveles, M. Marks, E. Marouzet, S. Marsh, B. Marshall, M. Marshall, J. Martin, A. Martineau, L.M. Martinez, N. Maskell, D. Matila, W. Matimba-Mupaya, L. Matthews, A. Mbuyisa, S. McAdoo, H. McAllister-Williams, A. McArdle, P. McArdle, D. McAulay, G.P. McCann, H.J.C. drury, J. McCormick, W. McCormick, P. McCourt, L. McGarvey, C. McGee, K. Mcgee, J. McGinness, K. McGlynn, A. McGovern, H. McGuinness, I.B. McInnes, J. McIntosh, E. McIvor, K. McIvor, L. McLeavey, A. McMahon, M.J. McMahon, L. McMorrow, T. Mcnally, M. McNarry, J. McNeill, A. McQueen, H. McShane, C. Mears, C. Megson, S. Megson, P. Mehta, J. Meiring, L. Melling, M. Mencias, D. Menzies, M. Merida Morillas, A. Michael, C. Miller, L. Milligan, C. Mills, N.L. Mills, L. Milner, S. Misra, J. Mitchell, A. Mohamed, N. Mohamed, S. Mohammed, P.L. Molyneaux, W. Monteiro, S. Moriera, A. Morley, L. Morrison, R. Morriss, A. Morrow, A.J. Moss, P. Moss, K. Motohashi, N. Msimanga, E. Mukaetova-Ladinska, U. Munawar, J. Murira, U. Nanda, H. Nassa, M. Nasseri, A. Neal, R. Needham, P. Neill, S. Neubauer, D.E. Newby, H. Newell, T. Newman, A. Newton-Cox, T. Nicholson, D. Nicoll, A. Nikolaidis, C.M. Nolan, M.J. Noonan, C. Norman, P. Novotny, J. Nunag, L. Nwafor, U. Nwanguma, J. Nyaboko, C. O'Brien, K. O'Donnell, D. O'Regan, L. O'Brien, N. Odell, G. Ogg, O. Olaosebikan, C. Oliver, Z. Omar, P.J.M. Openshaw, L. Orriss-Dib, L. Osborne, R. Osbourne, M. Ostermann, C. Overton, J. Owen, J. Oxton, J. Pack, E. Pacpaco, S. Paddick, S. Painter, A. Pakzad, S. Palmer, P. Papineni, K. Paques, K. Paradowski, M. Pareek, D. Parekh, H. Parfrey, C. Pariante, S. Parker, M. Parkes, J. Parmar, S. Patale, B. Patel, M. Patel, S. Patel, D. Pattenadk, M. Pavlides, S. Payne, L. Pearce, J.E. Pearl, D. Peckham, J. Pendlebury, Y. Peng, C. Pennington, I. Peralta, E. Perkins, Z. Peterkin, T. Peto, N. Petousi, J. Petrie, P. Pfeffer, J. Phipps, J. Pimm, K. Piper Hanley, R. Pius, H. Plant, S. Plein, T. Plekhanova, M. Plowright, K. Poinasamy, O. Polgar, L. Poll, J.C. Porter, J. Porter, S. Portukhay, N. Powell, A. Prabhu, J. Pratt, A. Price, C. Price, C. Price, D. Price, L. Price, L. Price, A. Prickett, J. Propescu, S. Prosper, S. Pugmire, S. Quaid, J. Quigley, J. Quint, H. Qureshi, I.N. Qureshi, K. Radhakrishnan, N.M. Rahman, M. Ralser, B. Raman, A. Ramos, H. Ramos, J. Rangeley, B. Rangelov, L. Ratcliffe, P. Ravencroft, A. Reddington, R. Reddy, H. Redfearn, D. Redwood, A. Reed, M. Rees, T. Rees, K. Regan, W. Reynolds, C. Ribeiro, A. Richards, E. Richardson, M. Richardson, P. Rivera-Ortega, K. Roberts, E. Robertson, E. Robinson, L. Robinson, L. Roche, C. Roddis, J. Rodger, A. Ross, G. Ross, J. Rossdale, A. Rostron, A. Rowe, A. Rowland, J. Rowland, M.J. Rowland, S.L. Rowland-Jones, K. Roy, M. Roy, I. Rudan, R. Russell, E. Russell, G. Saalmink, R. Sabit, E.K. Sage, T. Samakomva, N. Samani, C. Sampson, K. Samuel, R. Samuel, A. Sanderson, E. Sapey, D. Saralaya, J. Sargant, C. Sarginson, T. Sass, N. Sattar, K. Saunders, R.M. Saunders, P. Saunders, L.C. Saunders, H. Savill, W. Saxon, A. Sayer, J. Schronce, W. Schwaeble, J.T. Scott, K. Scott, N. Selby, M.G. Semple, M. Sereno, T.A. Sewell, A. Shah, K. Shah, P. Shah, M. Shankar-Hari, M. Sharma, C. Sharpe, M. Sharpe, S. Shashaa, A. Shaw, K. Shaw, V. Shaw, A. Sheikh, S. Shelton, L. Shenton, K. Shevket, A. Shikotra, J. Short, S. Siddique, S. Siddiqui, J. Sidebottom, L. Sigfrid, G. Simons, J. Simpson, N. Simpson, A. Singapuri, C. Singh, S. Singh, S.J. Singh, D. Sissons, J. Skeemer, K. Slack, A. Smith, D. Smith, S. Smith, J. Smith, L. Smith, M. Soares, T.S. Solano, R. Solly, A.R. Solstice, T. Soulsby, D. Southern, D. Sowter, M. Spears, L.G. Spencer, F. Speranza, L. Stadon, S. Stanel, N. Steele, M. Steiner, D. Stensel, G. Stephens, L. Stephenson, M. Stern, I. Stewart, R. Stimpson, S. Stockdale, J. Stockley, W. Stoker, R. Stone, W. Storrar, A. Storrie, K. Storton, E. Stringer, S. Strong-Sheldrake, N. Stroud, C. Subbe, C.L. Sudlow, Z. Suleiman, C. Summers, C. Summersgill, D. Sutherland, D.L. Sykes, R. Sykes, N. Talbot, A.L. Tan, L. Tarusan, V. Tavoukjian, A. Taylor, C. Taylor, J. Taylor, A. Te, H. Tedd, C.J. Tee, J. Teixeira, H. Tench, S. Terry, S. Thackray-Nocera, F. Thaivalappil, B. Thamu, D. Thickett, C. Thomas, D.C. Thomas, S. Thomas, A.K. Thomas, T. Thomas-Woods, T. Thompson, A.A.R. Thompson, T. Thornton, M. Thorpe, R.S. Thwaites, J. Tilley, N. Tinker, G.F. Tiongson, M. Tobin, J. Tomlinson, C. Tong, M. Toshner, R. Touyz, K.A. Tripp, E. Tunnicliffe, A. Turnbull, E. Turner, S. Turner, V. Turner, K. Turner, S. Turney, L. Turtle, H. Turton, J. Ugoji, R. Ugwuoke, R. Upthegrove, J. Valabhji, M. Ventura, J. Vere, C. Vickers, B. Vinson, E. Wade, P. Wade, L.V. Wain, T. Wainwright, L.O. Wajero, S. Walder, S. Walker, S. Walker, E. Wall, T. Wallis, S. Walmsley, J.A. Walsh, S. Walsh, L. Warburton, T.J.C. Ward, K. Warwick, H. Wassall, S. Waterson, E. Watson, L. Watson, J. Watson, J. Weir McCall, C. Welch, H. Welch, B. Welsh, S. Wessely, S. West, H. Weston, H. Wheeler, S. White, V. Whitehead, J. Whitney, S. Whittaker, B. Whittam, V. Whitworth, A. Wight, J. Wild, M. Wilkins, D. Wilkinson, B. Williams, N. Williams, N. Williams, J. Williams, S.A. Williams-Howard, M. Willicombe, G. Willis, J. Willoughby, A. Wilson, D. Wilson, I. Wilson, N. Window, M. Witham, R. Wolf-Roberts, C. Wood, F. Woodhead, J. Woods, D.G. Wootton, J. Wormleighton, J. Worsley, D. Wraith, C. Wrey Brown, C. Wright, L. Wright, S. Wright, J. Wyles, I. Wynter, M. Xu, N. Yasmin, S. Yasmin, T. Yates, K.P. Yip, B. Young, S. Young, A. Young, A.J. Yousuf, A. Zawia, L. Zeidan, B. Zhao, B. Zheng, O. Zongo

**Affiliations:** a National Heart and Lung Institute, Imperial College London, UK; b NIHR Health Protection Research Unit in Emerging and Zoonotic Infections, Institute of Infection, Veterinary and Ecological Sciences, University of Liverpool, UK; c MRC-University of Glasgow Centre for Virus Research, Immunity and Inflammation, University of Glasgow, UK; d Cardiovascular Research Team, Imperial College Healthcare NHS Trust, London, UK; e Department of Infection, Immunity and Cardiovascular Disease, University of Sheffield, Sheffield, UK; f Centre for Medical Informatics, The Usher Institute, University of Edinburgh, Edinburgh, UK; g University of Dundee, Ninewells Hospital and Medical School, Dundee, UK; h MRC Human Immunology Unit, University of Oxford, Oxford, UK; i Division of Infection, Immunity & Respiratory Medicine, Faculty of Biology, Medicine and Health, University of Manchester, Manchester, UK; j Radcliffe Department of Medicine, University of Oxford, Oxford, UK; k Asthma and Lung UK, London, UK; l Department of Clinical Research, London School of Hygiene & Tropical Medicine, London, UK; m Oxford Centre for Global Health Research, University of Oxford, Oxford, UK; n Institute for Lung Health, Leicester NIHR Biomedical Research Centre, University of Leicester, Leicester, UK; o Department of Population Health Sciences, University of Leicester, Leicester, UK; p Centre for Inflammation Research, University of Edinburgh, Edinburgh, UK; q The Pandemic Institute, University of Liverpool, UK

**Keywords:** COVID-19, SARS-CoV-2 immunity, Convalescent, Nasal antibody, Mucosal immunity, Vaccination, SARS-CoV-2 variants

## Abstract

**Background:**

Most studies of immunity to SARS-CoV-2 focus on circulating antibody, giving limited insights into mucosal defences that prevent viral replication and onward transmission. We studied nasal and plasma antibody responses one year after hospitalisation for COVID-19, including a period when SARS-CoV-2 vaccination was introduced.

**Methods:**

In this follow up study, plasma and nasosorption samples were prospectively collected from 446 adults hospitalised for COVID-19 between February 2020 and March 2021 via the ISARIC4C and PHOSP-COVID consortia. IgA and IgG responses to NP and S of ancestral SARS-CoV-2, Delta and Omicron (BA.1) variants were measured by electrochemiluminescence and compared with plasma neutralisation data.

**Findings:**

Strong and consistent nasal anti-NP and anti-S IgA responses were demonstrated, which remained elevated for nine months (*p* < 0.0001). Nasal and plasma anti-S IgG remained elevated for at least 12 months (*p* < 0.0001) with plasma neutralising titres that were raised against all variants compared to controls (*p* < 0.0001). Of 323 with complete data, 307 were vaccinated between 6 and 12 months; coinciding with rises in nasal and plasma IgA and IgG anti-S titres for all SARS-CoV-2 variants, although the change in nasal IgA was minimal (1.46-fold change after 10 months, *p* = 0.011) and the median remained below the positive threshold determined by pre-pandemic controls. Samples 12 months after admission showed no association between nasal IgA and plasma IgG anti-S responses (*R* = 0.05, *p* = 0.18), indicating that nasal IgA responses are distinct from those in plasma and minimally boosted by vaccination.

**Interpretation:**

The decline in nasal IgA responses 9 months after infection and minimal impact of subsequent vaccination may explain the lack of long-lasting nasal defence against reinfection and the limited effects of vaccination on transmission. These findings highlight the need to develop vaccines that enhance nasal immunity.

**Funding:**

This study has been supported by ISARIC4C and PHOSP-COVID consortia. ISARIC4C is supported by grants from the 10.13039/501100000272National Institute for Health and Care Research and the 10.13039/501100000265Medical Research Council. Liverpool Experimental Cancer Medicine Centre provided infrastructure support for this research. The PHOSP-COVD study is jointly funded by 10.13039/100014013UK Research and Innovation and National Institute of Health and Care Research. The funders were not involved in the study design, interpretation of data or the writing of this manuscript.

**Research in context**

**Evidence before this study**

While systemic immunity to SARS-CoV-2 is important in preventing severe disease, mucosal immunity prevents viral replication at the point of entry and reduces onward transmission. We searched PubMed with search terms “mucosal”, “nasal”, “antibody”, “IgA”, “COVID-19”, “SARS-CoV-2”, “convalescent” and “vaccination” for studies published in English before 20th July 2022, identifying three previous studies examining the durability of nasal responses that generally show nasal antibody to persist for 3–9 months. However, these studies were small or included individuals with mild COVID-19. One study of 107 care-home residents demonstrated increased salivary IgG (but not IgA) after two doses of mRNA vaccine, and another examined nasal antibody responses after infection and subsequent vaccination in 20 cases, demonstrating rises in both nasal IgA and IgG 7–10 days after vaccination.

**Added value of this study**

Studying 446 people hospitalised for COVID-19, we show durable nasal and plasma IgG responses to ancestral (B.1 lineage) SARS-CoV-2, Delta and Omicron (BA.1) variants up to 12 months after infection. Nasal antibody induced by infection with pre-Omicron variants, bind Omicron virus *in vitro* better than plasma antibody. Although nasal and plasma IgG responses were enhanced by vaccination, Omicron binding responses did not reach levels equivalent to responses for ancestral SARS-CoV-2. Using paired plasma and nasal samples collected approximately 12 months after infection, we show that nasal IgA declines and shows a minimal response to vaccination whilst plasma antibody responses to S antigen are well maintained and boosted by vaccination.

**Implications of all the available evidence**

After COVID-19 and subsequent vaccination, Omicron binding plasma and nasal antibody responses are only moderately enhanced, supporting the need for booster vaccinations to maintain immunity against SARS-CoV-2 variants. Notably, there is distinct compartmentalisation between nasal IgA and plasma IgA and IgG responses after vaccination. These findings highlight the need for vaccines that induce robust and durable mucosal immunity.

## Introduction

Intramuscular (i.m.) vaccines are remarkably effective in preventing severe COVID-19, their use being associated with declining hospitalisation.^1^^,^^2^ However, current vaccines provide only transient protection against respiratory viral replication, onward transmission and continuing emergence of variants.^3^, ^4^, ^5^ By contrast, respiratory infection with SARS-CoV-2 induces mucosal immune defences that can inhibit viral replication and transmission, though the correlation between nasal and systemic immunity is inexact.^6^^,^^7^ To date, there have been few studies of long-term nasal antibody durability and those that exist have studied relatively small groups, giving diverse results – suggesting that nasal antibody may persist for anywhere between 3 and 9 months.^8^, ^9^, ^10^ There is a clear need for additional studies of mucosal and systemic immunity in those recovered from severe disease.

Although i.m. vaccination transiently reduces transmission, vaccinees with breakthrough infections have peak nasopharyngeal viral loads similar to those in unvaccinated individuals.^4^^,^^5^ Some studies have shown that viral loads decline more rapidly in vaccinees,^5^ but it is unclear whether this effect is mediated by passive transudation of plasma antibody into the mucosa, or whether vaccination can recall mucosal responses primed by infection (as observed after i.m. influenza vaccination following an intranasal (i.n.) priming).^11^ Serum IgA and IgG is mostly monomeric and produced in the bone marrow, whereas nasal IgA is polymeric and can be synthesized locally by mucosal plasma cells.^12^^,^^13^ It is polymeric nasal IgA that is critical for efficient neutralisation of virus in the upper respiratory tract, and so passive transudation of plasma antibody into the mucosa is unlikely to provide durable sterilizing immunity.^6^^,^^12^ Understanding whether i.m. vaccination after COVID-19 can recall nasal IgA responses is an important step towards developing vaccines which prevent infection and transmission.

During worldwide circulation of SARS-CoV-2, multiple successive variants have evolved, driven by enhancements in transmissibility as well as immune evasion. The Omicron subvariants appear less susceptible to vaccine-induced immunity and show high reinfection rates.^14^^,^^15^ It seems that immunity induced by successive infection and vaccination may provide superior protection against Omicron compared with either alone^16^^,^^17^; and vaccination regimes which combine i.n and i.m. administration in mice induce enhanced mucosal protection against SARS-CoV-2 variants.^18^ This suggests that priming the nasal mucosa is required to induce effective local antibody responses that might provide enhanced immunity against current and future variants. However, the cross-reactivity of nasal antibody after infection with pre-Omicron virus is unknown.

We here report the results of a large multicentre follow-up study of nasal and plasma antibody responses approximately a year after COVID-19, aiming to understand the longevity of nasal antibody responses after COVID-19 and the effect of subsequent vaccination. We demonstrate durable nasal and plasma IgG responses to ancestral (B.1 lineage) SARS-CoV-2, Delta and Omicron variant that are enhanced by i.m. vaccination. However, nasal IgA responses did not mirror those in plasma, waned after 9 months and were not substantially boosted by vaccination (Fig. S1).

## Methods

### Study design and ethics

Clinical data, nasosorption and plasma samples were collected from hospitalised cases of COVID-19 within the ISARIC4C and PHOSP-COVID multicentre studies of UK adult patients (Fig. S2).^19^^,^^20^

Adults hospitalised during the SARS-COV-2 pandemic were systematically recruited into the International Severe Acute Respiratory and Emerging Infection Consortium (ISARIC) World Health Organization Clinical Characterisation Protocol UK study (IRAS260007 and IRAS126600).^20^ Written informed consent was obtained from all patients. Ethical approval was given by the South Central–Oxford C Research Ethics Committee in England (reference: 13/SC/0149), Scotland A Research Ethics Committee (20/SS/0028) and World Health Organization Ethics Review Committee (RPC571 and RPC572l; 25 April 2013).

After hospital discharge patients >18 years old who had no co-morbidity resulting in a prognosis of less than 6 months, were recruited to the PHOSP-COVID study. Both sexes were recruited and gender was self-reported. Written informed consent was obtained from all patients. Ethical approvals for the PHOSP-COVID study were given by Leeds West Research Ethics Committee (20/YH/0225).

Control samples were collected from healthy volunteers without respiratory disease or symptoms of infection prior to the COVID-19 pandemic. Written consent was obtained for all individuals and ethical approvals were given by London-Harrow Research Ethics Committee (13/LO/1899).

Samples were collected on day 1–9 of admission and/or at intervals during convalescence (approximately 1–14 months after discharge). Clinical data were collected to account for variables which may affect antibody titre, via hospital records and self-reporting (Table 1). Where missing, vaccination data were accessed through linkage between NHS digital data and the ISARIC4C study within the Outbreak Data Analysis Platform (ODAP). Disease severity was classified according to the WHO Clinical Progression score.^21^

**Table 1:** Summary of clinical and demographic data.

Demographics (n = 446)			Missing data
Age at admission, years		59 (51–67)	28 (6.3)
Sex at birth	Female	164 (39.1)	27 (6.0)
	Male	255 (60.9)	
Ethnicity	White	259 (82.0)	130 (29.0)
	South Asian	22 (6.9)	
	Black	20 (6.3)	
	Mixed	5 (1.6)	
	Other	10 (3.2)	
Clinical characteristics (n = 446)			Missing data
Disease severity	WHO Class 3-4	60 (14.6)	34 (7.6)
	WHO Class 5	193 (46.8)	
	WHO Class 6	101 (24.5)	
	WHO Class 7-9	48 (11.6)	
	WHO Class 10	10 (2.4)	
BMI ≥30		164 (62.4)	183 (41.0)
Co-morbidities	None	66 (20.8)	128 (28.7)
	1	69 (21.8)	
	≥2	183 (57.4)	
First vaccination received during the study	Yes	307 (95.0)	123 (27.6)
	No	16 (5.0)	
Second vaccination received during the study	Yes	225 (78.9)	161 (36.1)
	No	60 (21.1)	
Type of first vaccination	Oxford/AstraZeneca (ChAdOx1 nCoV-19)	185 (59.9)	137 (30.7)
	Pfizer/Bio-N-Tec (BNT162b2)	124 (40.1)	
	Moderna	0	
Type of second vaccination	Oxford/AstraZeneca (ChAdOx1 nCoV-19)	89 (62.7)	304 (68.1)
	Pfizer/Bio-N-Tec (BNT162b2)	51 (35.9)	
	Moderna	2 (1.4)	

Data are n (%) or median (IQR). Percentages were calculated after exclusion of missing data. Disease severity is classified according to the WHO Clinical Progression score: 3–4 = no continuous supplemental oxygen needed; 5 = continuous supplemental oxygen only; 6 = continuous or bi-level positive airway pressure ventilation or high-flow nasal oxygen; 7–9 = invasive mechanical ventilation or other organ support; and 10 = did not survive. BMI = body-mass index.

### Procedures

Nasal fluid was collected using a NasosorptionTM FX·I device (Hunt Developments UK Ltd), which uses a synthetic absorptive matrix to collect concentrated nasal fluid. Samples were stored and eluted as previously described, with the addition of 1% (final v/v) Triton-X to elution buffers to inactivate SARS-CoV-2.^22^ EDTA plasma was collected from whole blood taken by venepuncture (full details in the supplement).

### Immunoassays

Antibody responses were measured by MSD electrochemiluminescence multiplex assay (Mesoscale Diagnostics, Rockville, Maryland, USA). Nasal and plasma IgA and IgG responses to Spike (S), Nucleocapsid (NP) and the Receptor-Binding-Domain of Spike (RBD) antigens of ancestral (B.1 lineage) SARS-CoV-2 were measured using MSD V-PLEX COVID-19 Coronavirus Panel 2 Kits (IgG Cat No: K15369U-4; IgA Cat No: K15371U-4), which have been demonstrated to have excellent sensitivity and specificity.^23^ All reagents used were kit specific and provided by MSD. Nasal and plasma antibody responses to RBD antigen of Delta (AY3; AY4; AT4.2; AY5:AY6; AY.7; AY.12; AY.14; B.1.617.2) and Omicron (B.1.1.529; BA.1) variants were measured using MSD V-PLEX SARS-CoV-2 panel 22 (IgG Cat No: K15559U-4; IgA Cat No: K15561U-4). The MSD plates consisted of 96 wells each containing 10 pre-coated antigen spots. BSA served as a negative control in each well. Plasma was analysed at a fixed dilution of 1 in 5000, using MSD Diluent 100 as per the manufacturers protocol.^24^ The composition of nasal fluid is more variable than plasma. Thus, a fixed dilution of 1 in 50 was chosen after a serial dilution experiment which measured virus-specific IgA and IgG concentration in acute, convalescent and pre-pandemic nasal fluid samples, confirming the validity and reproducibility of this method. To enhance the accuracy and reproducibility of results, total IgA and IgG concentration in each nasal sample was measured and antibody titre was normalised (see ‘Statistics’).

Plates were blocked with MSD blocker A, prior to sample analysis to prevent non-specific binding. Diluted samples were incubated followed by addition of MSD SULFO-TAG Anti-Human IgA or IgG antibody to detect bound immunoglobulin. Plates were subsequently measured on a MESO QuickPlex SQ 120 Reader (MSD). An equivalent assay for responses to RBD from ancestral (B.1 lineage) SARS-CoV-2 was present on panel 2 and panel 22 to ensure comparable performance between kits. Antibody concentrations were calculated using a reference standard of convalescent plasma and assigned arbitrary units (AU/mL). All values at or below the lower limit of detection (LLOD) were replaced with LLOD. All values at or above the upper limit of detection (ULOD) were replaced with ULOD.

Total IgA and IgG content of nasal fluid was measured using a human antibody isotyping Procarta-Plex protein quantitation immunoassay (Invitrogen, Massachusetts, United States, Cat No.: EPX070-10818-901). Nasal samples analysed at a fixed dilution of 1 in 50. Plates were prepared according to the manufacturers protocol (Publication Number MAN0024721)^25^ and read on a BioPlex200 instrument (Bio-Rad, UK). All values at or below the lower limit of detection (LLOD) were replaced with LLOD. All values at or above the upper limit of detection (ULOD) were replaced with ULOD.

Neutralising activity in each plasma sample was measured by a serial dilution approach.^26^ Each sample was serially diluted in triplicate from 1:50 to 1:36,450 in complete DMEM prior to incubation with HIV (SARS-CoV-2) pseudotypes, incubated for 1 h, and plated onto 239-ACE2 target cells. After 48–72 h, luciferase activity was quantified by the addition of Steadylite Plus chemiluminescence substrate and analysis on a PerkinElmer EnSight multimode plate reader (PerkinElmer, Beaconsfield, UK). Antibody titre was then estimated by interpolating the point at which infectivity had been reduced to 90% of the value for the no serum control samples (full details in the supplement).

### Statistics

Analyses were conducted on ODAP. All tests were two-tailed and statistical significance was defined as a *p*-value < 0.05 after adjustment for false discovery rate (q-value = 0.05). Sample size calculations are detailed in the supplement.

Nasal virus-specific IgA and IgG (AU/mL) was normalised to total IgA or IgG concentration in each sample, respectively (pg/mL). This accounted for variability in concentration of sample obtained and matrix effects. Plasma and normalised nasal data were log_2_ transformed prior to all analyses. The data were confirmed to be non-parametrically distributed using quantile vs quantile plots. To understand the durability of antibody responses, comparisons between timepoints were made using the optimal pooled t-test, which performs well in non-parametric partially paired data.^27^ All missing clinical data were excluded, except where date of symptom onset was missing, in which case time from symptom onset was approximated according to the visit number and the date of admission, if known. To estimate the effect of vaccination on antibody trajectories, a LOESS regression curve was fitted to data from repeated and cross-sectional samples taken from those who were known to be vaccinated.

To explore the relationship between plasma and nasal responses variables were analysed in a correlation matrix, measuring the Spearman rank correlation coefficient between variables. Disease severity and age were included as co-variates. Variables were scaled and centred prior to analysis. The variables in the correlogram were hierarchically clustered using Ward's minimum variance, minimising the Euclidian distance between variables.

To further explore the factors determining the relationship between nasal IgA and plasma responses after vaccination, unsupervised clustering was performed using hierarchical clustering with Ward's minimum variance, minimising the Euclidian distance between individuals who had samples taken after 10 months. For any paired or repeated measures within the time frame, the latter of the samples was selected for analysis. Variables were scaled and centred prior to analysis. Heatmap rows were annotated with WHO clinical progression score and age to determine if either factor associated with clusters. The number of clusters was determined using the Silhouette score. To understand how vaccination might affect cluster membership, the mean time from vaccination was compared between each cluster using the Kruskal-wallis test. Co-variates were not included in the LOESS regression or clustering analysis. The proportion of individuals in each cluster receiving ChAdOx1 nCoV-19 vaccine was compared using the chi-squared test. Analyses were undertaken and visualised using the ‘cluster’, ‘ggplot2’, ‘corrplot’, ‘ggpubr’, ‘ggstatsplot’, ‘factoextra’ and ‘pheatmap’ package in R version 4.0.5.

Control samples were used to define a nasal antibody threshold. The threshold was equivalent to mean + 2 SD of log2-transformed antibody data, and validated against standardized WHO BAU/mL thresholds converted into MSD AU/mL.^28^

The raw data underlying this study are available upon reasonable request. Please see the Data sharing statement.

### Role of the funders

ISARIC4C is supported by grants from the 10.13039/501100000272National Institute for Health and Care Research (award CO-CIN-01) and the 10.13039/501100000265Medical Research Council (grant MC_PC_19059) Liverpool Experimental Cancer Medicine Centre provided infrastructure support for this research (grant reference: C18616/A25153). The PHOSP-COVD study is jointly funded by 10.13039/100014013UK Research and Innovation and 10.13039/501100000272National Institute for Health and Care Research (grant references: MR/V027859/1 and COV0319). The funders were not involved in the study design, interpretation of data or the writing of this manuscript.

## Results

A total of 446 adults, hospitalised between February 2020 and March 2021 were recruited, of which 141 provided samples at sequential time points. 569 plasma samples were collected, of which 338 represented samples taken at sequential timepoints. In addition, 356 nasal samples were collected, of which 143 were from sequential timepoints. 174 individuals had paired plasma and nasal samples taken at a given time point. Characteristics of the patients providing sequential samples and the total cohort were comparable (Table 1 and S1). The 6 and 12 month samples were collected between November 2020 and March 2022, covering the start of the UK vaccination campaign (Fig. S2).

### Plasma antibody responses are more durable than nasal responses after COVID-19

**Fig. 1:**
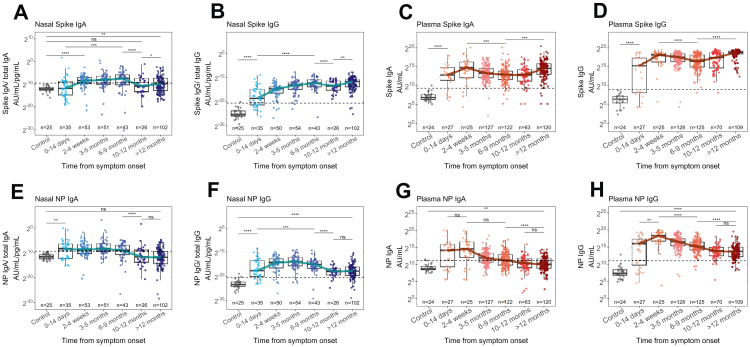
**Nasal and plasma antibody responses 12 months after infection**. Nasal (A,B) and plasma (C,D) IgA and IgG responses to S of ancestral SARS-CoV-2 from 446 COVID-19 patients, compared to 25 pre-pandemic control samples (grey). Nasal (E,F) and plasma (G,H) IgA and IgG responses to NP of ancestral SARS-CoV-2. Nasal virus-specific antibody titres were normalised to total IgA or IgG concentration. Blue and red lines indicate the trajectory of median titres across timepoints. The horizontal dashed line indicates the threshold for positivity determined by the mean+2SD of controls. ∗ = *p* < 0.05, ∗∗ = *p* < 0.01, ∗∗∗ = *p* < 0.001, ∗∗∗∗ = *p* < 0.0001.

Nasal anti-S and anti-NP IgA appeared within 4 weeks after symptom onset but waned after 9 months to levels equivalent to pre-pandemic controls (*p* < 0.0001, Wilcoxon test) (Fig. 1). Anti-S IgG appeared within 14 days of symptom onset (*p* < 0.0001, Wilcoxon test) and rose 2181-fold after 9 months (*p* < 0.0001, pooled t-test) but unlike IgA responses, remained above pre-pandemic controls thereafter (*p* < 0.0001, Wilcoxon test) (Fig. 1A and B). Both nasal IgA and IgG anti-S titres rose after 10 months, though the median change was only 1.46-fold in the case of IgA (*p* = 0.011, pooled t-test). Anti-NP IgA and IgG responses remained low after 9 months (*p* < 0.0001, pooled t-test) (Fig. 1E and F).

Pre-pandemic controls allowed a threshold value for nasal antibody to be established, equivalent to the mean+2SD (Fig. 1). Applying the same method to plasma samples, we found that the threshold was similar, though more conservative than the WHO threshold, confirming the validity of the method (Fig. S3). Using this threshold, we found that the median nasal IgA response to S and NP fell and remained below threshold after 9 months (Fig. 1A and E) whilst the median nasal IgG titre was durable and remained above threshold for both antigens at 12 months (Fig. 1B and F).

Plasma IgG anti-S and anti-NP responses developed within 14 days of symptom onset and remained elevated after 12 months (*p* < 0.0001, pooled t-test) (Fig. 1D and H). Notably, the trajectories of plasma IgA and IgG responses differed to that of nasal IgA. Whilst nasal responses peaked between 6 and 9 months for S and between 3 and 5 months for NP, plasma responses peaked within 4 weeks before waning (Fig. 1). Notably, plasma anti-NP responses plateaued after 10 months and most patients remained seropositive for both antigens at the final time point, indicating durable plasma responses after COVID-19 (Fig. 1D and H).

Only 2 of 446 individuals showed serological evidence of reinfection (whereby a rise in both anti-NP and anti-S IgG was observed between 103 and 308 days after infection for the first individual and between 238 and 463 days for the second). Furthermore, in 61 individuals where vaccination status was known and from whom paired samples were taken before and after vaccination, anti-S titres rose (*p* < 0.0001, paired Wilcoxon test) whilst anti-NP titres declined (*p* < 0.0001, paired Wilcoxon test), as expected, indicating a low prevalence of re-infection in our cohort (Fig. S4A and B). These data therefore demonstrate that nasal and plasma IgG responses are durable after COVID-19, whilst nasal IgA responses last only 9 months.

### Responses during vaccination campaign

Of those with known vaccination status (n = 323), 95.0% received their first SARS-CoV-2 vaccination during the study. Vaccinations occurred between December 2020 and December 2021. Of these, 59.9% received ChAdOx1 nCoV-19 as the first dose (Table 1). The median date of vaccination was 13th February 2021 (IQR Jan–March 2021) and samples taken after 12 months were collected between March 2021 and March 2022 (median 20th June 2021). Therefore, most samples taken after 12 months were taken after vaccination. Vaccines only induce responses against S protein, making vaccination the most likely cause of rises seen in nasal anti-S IgG (and to a lesser extent IgA) responses after 12 months, given that nasal anti-NP responses declined during this time (Fig. 1).

**Fig. 2:**
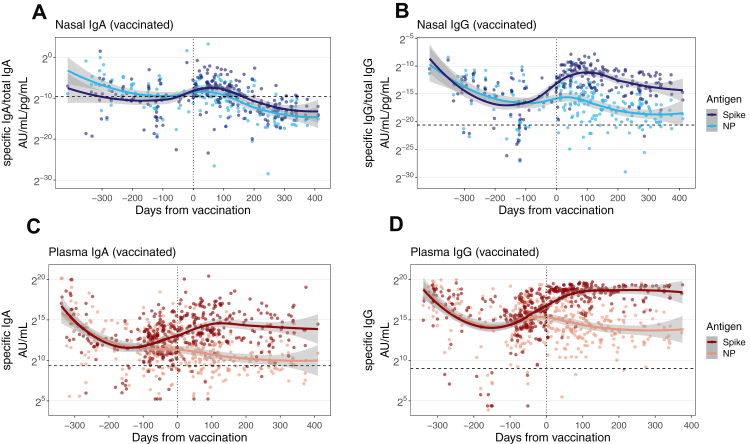
**Nasal and plasma antibody trajectories in vaccinated individuals**. Trajectory of nasal IgA and IgG responses from 120 COVID-19 patients before and after first vaccination (A–B). Plasma IgA and IgG responses from 323 COVID-19 patients before and after first vaccination (C–D). Trajectories were modelled using a LOESS regression curve and 95% confidence intervals are shown in grey. The vertical dotted line indicates the time of first vaccination. The horizontal dashed line indicates the threshold for positivity determined by the mean+2SD of controls.

We confirmed the effect of vaccination by comparing nasal S and NP antibody responses in individuals known to be vaccinated during the study (n = 120) (Fig. 2). There were clear differences in the nasal IgA and IgG responses after vaccination (Fig. 2A–B). Although nasal anti-S IgA responses appeared to transiently rise after vaccination, there was no difference between the trajectories of the anti-NP and anti-S responses and the 95% CIs overlapped. By contrast, nasal IgG anti-S responses rose substantially after vaccination and peaked approximately 100 days after vaccination, whilst the anti-NP trajectory declined. There was no overlap between the 95% confidence intervals (CI) of the regression curve for anti-S and anti-NP IgG responses after vaccination indicating distinct trajectories (Fig. 2B). Notably, the nasal IgG responses mirrored that of plasma IgA and IgG (Fig. 2C–D). Thus, in keeping with our previous analysis (Fig. 1), changes in nasal IgA titres after vaccination are minor compared to nasal and plasma IgG which appear substantially boosted. These findings suggest that vaccination may not fully recall mucosal IgA responses.

### Responses to Delta and Omicron (BA.1) variants

**Fig. 3:**
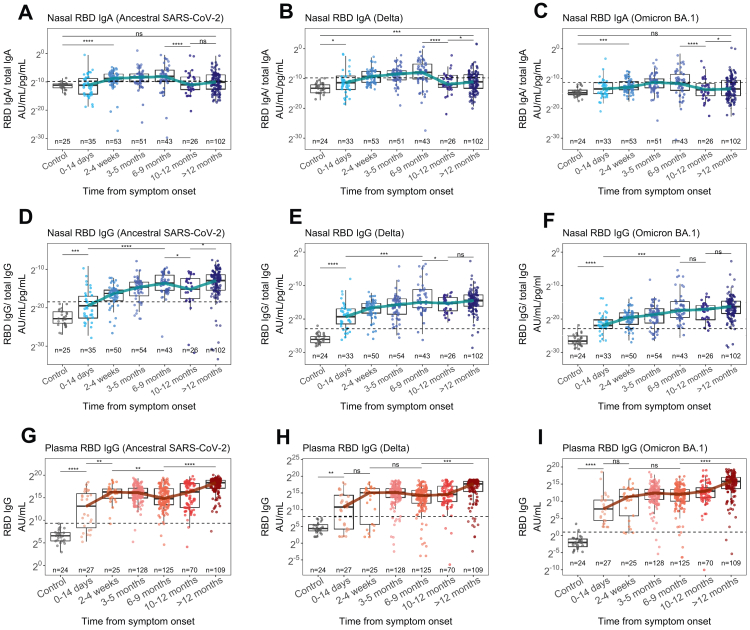
**Nasal and plasma antibody responses to variants of concern 12 months after infection**. Nasal IgA (A–C), nasal IgG (D–G) and plasma IgG (G–I) responses to RBD of ancestral SARS-CoV-2, Delta, and Omicron (BA.1) variants in 446 COVID-19 patients compared to 25 pre-pandemic control samples (grey). Nasal virus-specific antibody titres were normalised to total IgA or IgG concentration. The horizontal dashed line indicates the threshold for positivity determined by the mean+2SD of controls. ∗ = *p* < 0.05, ∗∗ = *p* < 0.01, ∗∗∗ = *p* < 0.001, ∗∗∗∗ = *p* < 0.0001.

All participants were admitted to hospital prior to the emergence of Omicron variant and 71.1% (n = 317) were admitted before 10th May 2021 when Delta variant became the dominant strain in the UK.^4^^,^^14^ However, nasal IgA and IgG responses binding both Delta and Omicron RBD were present within 28 days of symptom onset and remained elevated for at least 9 months (Fig. 3A–F). Nasal IgA binding Omicron appeared the most short-lived; while titres were above pre-pandemic controls between 2 and 4 weeks, the median titre only passed the threshold for positivity between 3 and 5 months post-infection (Fig. 3C). Furthermore, at its peak, Omicron binding nasal IgA was only 10-fold above controls (*p* < 0.0001), compared to nasal IgA binding ancestral SARS-CoV-2 RBD which was 28-fold higher (*p* < 0.0001) (Fig. 3A and C). Plasma IgG responses to Delta and Omicron also developed within 14 days and were sustained for 12 months (Fig. 3G–I).

To understand the degree of cross-reactivity between compartments we compared the ratio of antibody binding RBD of Omicron virus and ancestral SARS-CoV-2 (Fig. S5). There was no difference in the median ratio between nasal IgA (0.10) and nasal IgG (0.12, *p* = 0.67, Wilcoxon test). However, the nasal IgG ratio was higher than that of plasma IgG (0.09, *p* = 0.020, Wilcoxon test) and the nasal IgA ratio was higher than that of plasma IgA (0.08, *p* = 0.00059, Wilcoxon test). These data indicate that infection with pre-Omicron SARS-CoV-2 can induce nasal and plasma antibody that binds Omicron RBD, and that nasal antibody may have greater cross-binding potential. However, despite this, Omicron-binding nasal IgA is slow to reach positive levels and is transiently maintained.

### Responses to Delta and Omicron (BA.1) variant after vaccination

The nasal IgA response to Delta and Omicron variant did not appear substantially different after vaccination (Fig. S6A) though a small rise in the median Omicron- and Delta-binding nasal IgA was seen after 12 months, when most individuals had been vaccinated (Fig. 3A–C). However, this difference was small and the median titre did not reach the positive threshold. Nasal IgG responses to Omicron and Delta variant rose after vaccination (Fig. S6B), although Omicron-binding responses did not reach the level of those to Delta and ancestral SARS-CoV-2 despite vaccination.

Plasma IgG responses to Delta and Omicron variants also rose after vaccination but did not reach levels of responses to ancestral virus (Fig. S6D). To study the effect of vaccination specifically and validate trends seen in cross-sectional data, we identified 61 individuals from whom paired pre- and post-vaccination plasma samples were collected; these were taken at a median of 54 days (IQR 25.6–68.8) before the first vaccination dose and 176 days (IQR 113–212) after (Fig. S4). Vaccination boosted both Omicron-binding (*p* < 0.0001) and Delta-binding plasma IgG (*p* = 0.0019) in most individuals. These data suggest that vaccination can boost Omicron- and Delta-binding nasal and plasma IgG responses but to a lesser degree than ancestral virus responses. Meanwhile, Omicron- and Delta-binding nasal IgA responses may not be affected by vaccination.

### Plasma neutralising antibody to SARS-CoV-2 variants

Plasma neutralising titres against ancestral, Delta and Omicron variants of SARS-CoV-2 remained substantially elevated compared with controls between 3 and 12 months (Fig. S7). However, neutralising titres against Omicron were generally lower: at 10–12 months, 76.2% had neutralising antibody against Omicron, compared to 92.5% against ancestral SARS-CoV-2. Neutralising titres against all three variants were boosted during the vaccination campaign (*p* < 0.0001, pooled t-test) indicating that i.m. vaccination after COVID-19 can enhance neutralising antibody levels to homologous and heterologous variants.

As expected, neutralising antibody titres correlated with plasma RBD (R = 0.82, *p* < 0.0001, Spearman's rank) and S IgG (R = 0.81, *p* < 0.0001, Spearman's rank) (Fig. S8). Notably, plasma neutralising antibody correlated with nasal anti-RBD IgG (R = 0.62, *p* < 0.0001, Spearman's rank) and anti-S IgG (R = 0.58, *p* < 0.0001, Spearman's rank) but not nasal IgA (anti-RBD R = 0.0035, *p* = 0.98, Spearman's rank). This finding, alongside the boosting of nasal IgG after vaccination indicate that nasal IgG responses reflect that of plasma, whilst the nasal IgA response is distinct and compartmentalised (Fig. 2 and S8).

### Discordance between plasma and nasal antibody responses

**Fig. 4:**
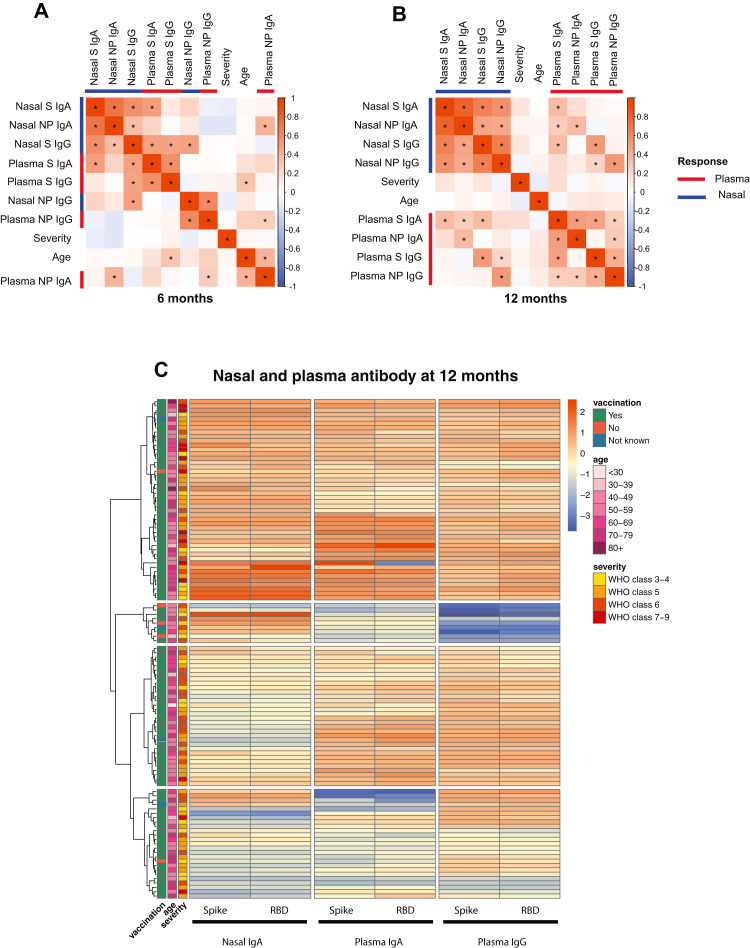
**Relationship between nasal and plasma antibody responses at 6 and 12 months**. A) Correlogram of nasal and plasma IgA and IgG responses to S and NP, disease severity and age at 6 months (n = 62), when 48 of 52 individuals with known vaccination status had received their first vaccination and B) correlogram at 12 months (n = 112), when 103 of 108 individuals with known vaccination status had been vaccinated. All statistically significant correlations (*p* < 0.05) are denoted with ∗. The variables were hierarchically clustered. C) Heatmap of nasal IgA, plasma IgA and plasma IgG responses to S and RBD at 12 months (n = 112). Rows are annotated with vaccination status, age and disease severity according to the WHO clinical progression score: 3–4 = no continuous supplemental oxygen needed; 5 = continuous supplemental oxygen only; 6 = continuous/bi-level positive airway pressure ventilation or high-flow nasal oxygen; 7–9 = invasive mechanical ventilation or other organ support.

To characterise the relationship between compartments, paired nasal and plasma samples from 174 individuals were examined. Samples were divided into those taken at approximately 6 months (3–9 months) and 12 months (>10–12 months) after infection (Fig. 4). At 6 months nasal anti-S IgA responses correlated strongly with nasal anti-NP IgA responses (R = 0.71, *p* < 0.0001, Spearman's rank) but showed a weaker association with nasal anti-S IgG (R = 0.57, *p* < 0.0001, Spearman's rank) and plasma anti-S IgA responses (R = 0.50, *p* < 0.0001, Spearman's rank) (Fig. 4A). There was no association between nasal IgA responses and plasma IgG response to either S (*p* = 0.38, Spearman's rank) or NP (*p* = 0.56, Spearman's rank). Nasal anti-NP IgA did not correlate with either nasal or plasma anti-NP IgG and correlated weakly with plasma anti-NP IgA (R = 0.40, *p* = 0.0021, Spearman's rank). Nasal IgG responses correlated with plasma IgG responses to the corresponding antigen (anti-S R = 0.47, *p* < 0.0001 and anti-NP R = 0.6, *p* < 0.0001, Spearman's rank). Compartmentalisation of nasal responses was even greater at 12 months when there was only weak association between nasal and plasma anti-S IgA (R = 0.35, *p* < 0.0001, Spearman's rank) (Fig. 4B).

Age and disease severity showed no association with nasal responses (Fig. 4B) and nasal responses were no different between male and female sex (Fig. S9A and B). Thus, we considered that the differences observed between plasma and nasal antibody responses were driven by vaccination. At 6 months, 48 of 52 individuals with known vaccination status had received their first vaccination and 27 had received both doses. The median time from first vaccination was 24 days (IQR 0.5–86). Meanwhile at 12 months, 103 of 108 individuals with known vaccination status had received their first vaccination and 75 had received both. The median time from last vaccination was 141 days (IQR 71–203). Thus, we reasoned that the increased compartmentalisation at 12 months may result from vaccination; whereby plasma responses are enhanced but nasal IgA is minimally and transiently affected.

To further explore the relationship between nasal IgA and plasma antibody responses after vaccination, we performed hierarchical clustering of anti-S/RBD responses from paired samples collected at 12 months. Compartmentalisation of nasal IgA from plasma responses was observed with 4 distinct clusters forming (Fig. 4C). The first cluster exhibited patients with strong nasal IgA and plasma responses. Patients with the weakest plasma IgA and IgG responses were present in cluster 2 whilst patients with the weakest nasal IgA responses were in cluster 3 and 4. Although not statistically significant, there was a tendency towards more recent vaccination in cluster 1 compared with cluster 3 and 4 (Fig. S9C). The date of vaccination was only available for two members of cluster 2. The proportion of individuals receiving BNT162b2 vaccination was similar across clusters (Fig. S9D) and there was no association between disease severity or age and cluster membership (Fig. 4C). Thus, we concluded that the clusters resulted from transient boosting of nasal IgA responses after recent vaccination, with divergence between the nasal IgA and plasma responses with increasing time from vaccination. Given the insubstantial and transient effect of vaccination on nasal IgA responses relative to plasma responses, we suggest that i.m. vaccination after COVID-19 does not recall mucosal responses.

## Discussion

We demonstrate durable nasal and plasma IgG responses to ancestral (B.1 lineage), Delta and Omicron variants of SARS-CoV-2 in 446 adults hospitalised with COVID-19, who were infected with pre-Omicron virus and the majority of whom were subsequently vaccinated. However, we found that nasal virus-specific IgA levels fell back to pre-COVID levels after 9 months and Omicron-binding nasal responses were particularly short-lived. Our results suggest that nasal IgA responses are compartmentalised from systemic responses after vaccination, which boosted nasal and plasma IgG but had limited effects on nasal IgA.

The durability of nasal antibody responses has hitherto been unclear. Whilst a Dutch study of healthcare workers found that nasal antibody lasted 9 months after mild infection, others demonstrated rapid waning after 3 months.^8^^,^^9^ Neither study examined a large cohort of hospitalised patients, and our findings confirm that COVID-19 can induce durable mucosal immunity. We also found that sex, disease severity and age did not impact the longevity of the nasal responses in keeping with a recent study of 26 unvaccinated individuals.^10^

By calibrating nasal antibody levels with pre-COVID samples, we demonstrate that on average, nasal IgA responses disappear after 9 months and Omicron-binding IgA is particularly short-lived. Nasal IgA is the most abundant mucosal antibody and provides an important first-line defence against respiratory infection. The importance of nasal IgA in mediating immunity to SARS-CoV-2 is highlighted by a recent study where nasal IgA but not IgG correlates with nasal neutralisation after COVID-19.^10^ The short-lived nasal IgA response demonstrated here may explain the high rates of infection with Omicron variant, despite vaccination, and are in-keeping with real-world data showing that infection with pre-Omicron virus has minimal influence on the risk of Omicron infection at 15 months.^15^^,^^29^

Whilst we found that i.m. vaccination can boost nasal IgG, our data suggest it has limited effects on IgA, in keeping with a previous study of salivary antibody in 107 care home residents.^30^ We demonstrated correlations between nasal IgG, plasma IgG and plasma neutralisation, whilst nasal IgA responses were compartmentalised, suggesting that the rise in nasal IgG after vaccination could derive from plasma. Notably, we demonstrate that those exhibiting stronger nasal IgA responses relative to plasma had been recently vaccinated. Although this analysis was limited by small sample size, our findings suggest that vaccination only transiently boosts nasal IgA. We did not observe differences in nasal IgA responses according to the type of vaccination received. mRNA vaccines tend to induce stronger circulating antibody responses than those using adenoviral vectors, but our results suggest this may not apply to nasal responses.^31^^,^^32^ Taken together, these findings suggest that i.m. vaccination after COVID-19 is unlikely to recall mucosal responses.

The concept of independent mucosal and systemic immunity is supported by recent studies showing that SARS-CoV-2 naïve individuals (whose mucosa have not been primed) do not produce nasal IgA after i.m. vaccination, highlighting that an independent response must occur at mucosal sites.^9^^,^^33^ Moreover, previous work has demonstrated that transudation of plasma antibody makes minimal contribution to total antibody concentrations in the mucosa, even in cases of paraproteinaemia where plasma concentrations are extremely high.^34^ This would explain why i.m. vaccination has had only transient effects on transmission,^4^ which may be mediated by transiently enhanced nasal IgG responses, which we demonstrate after vaccination. Given that monomeric IgG does not efficiently neutralise virus in the mucosa, it is unlikely that boosting this response will have considerable impact on mucosal susceptibility to infection.^6^^,^^10^ Future vaccines will need to substantially boost nasal IgA if they are to fully prevent infection and transmission. To date, intranasal and aerosolized vaccines have shown the most promise in doing so.^18^^,^^33^^,^^35^ It is therefore essential to prioritise development of mucosal vaccines which can provide better protection against respiratory infections.

### Study limitations

This large multicentre study has enabled confirmation of nasal antibody durability and its relationship to plasma responses. Our cohort was similar to the general population of adults discharged from hospital after COVID-19 in the UK.^20^ However, given the nature of this follow-up study it is likely that recruitment was biased towards patients who had recovered and were able to attend hospital visits during the convalescent phase.

Although 141 individuals had sequential samples taken after hospital discharge, given the circumstances and scale of this study we were not able to collect longitudinal samples from all participants. However, given that most individuals follow similar antibody kinetics, where longitudinal samples were missing, we approximated trajectories using cross-sectional samples taken from individuals in the acute and convalescent phases of illness.

We were not able to collect paired pre- and post-vaccination nasal samples in most individuals. However, we demonstrated clear differences in nasal anti-S and anti-NP responses after vaccination in 120 individuals, enabling inferences to be drawn. Whilst acquisition bias may have been introduced by this missingness, UK census data have indicated that 90% of the population had received one vaccine by August 2022,^36^ indicating that our cohort with complete data (in which 95% were vaccinated) are likely representative of the general population and that missing data were missing at random. Notably we estimated a peak of nasal anti-S IgG titres 100 days after vaccination which is considerably slower than peak circulating antibody responses after vaccination (28–42 days).^37^ Future studies using longitudinal data collected before and after vaccination will provide further insights into the effect of vaccination on nasal antibody trajectory.

Individuals in this study were not systematically screened for reinfection. However, this study was predominantly carried out during periods of national lockdown when incidence of infections was low.^38^ Furthermore, we analysed plasma taken from individuals for serological evidence of reinfection and found only 2 cases, suggesting that reinfection did not contribute to the trends we observed. Future studies examining the impact of reinfection on nasal IgA titres will provide further insight into memory formation in the mucosa.

Selective IgA deficiency (SIgAD) has been associated with increased risk of severe COVID-19 and we were unable to test patients for this condition. However, most individuals had persistently elevated plasma anti-S IgA responses during the study, suggesting that the prevalence of this condition was low. This is in keeping with one Turkish study of 424 COVID-19 patients which found only 11/424 had SIgAD.^39^ Given that Turkey has a higher incidence of SIgAD than the UK (0.52% in Turkey and 0.11% in the UK) we anticipate even lower prevalence in our study.^40^

### Conclusions

This study demonstrates durable but compartmentalised nasal IgA and plasma antibody responses to SARS-CoV-2 after infection and subsequent vaccination. We show enhancement of nasal and plasma IgG responses to ancestral SARS-CoV-2, Delta and Omicron variants after vaccination. However, nasal IgA responses, especially those to Omicron, are more short-lived and are not substantially affected by vaccination. Our results explain the lack of long-term sterilising immunity after previous infection and/or vaccination and highlight the need for mucosal vaccines that target nasal IgA responses. By enhancing nasal antibody responses, mucosal vaccines might prevent infection and transmission more effectively, enabling greater control of the pandemic and limiting the emergence of variants.

## Contributors

**FL** recruited participants, acquired clinical samples, analysed and interpreted the data and co-wrote this manuscript, including all drafting and revisions. **ST, AC, DS, JKS, CE, SCM and CD** contributed to acquisition of data underlying this study and have supported drafting and revisions of this work. **BJW, SS and NL** have made substantial contributions to the acquisition of data for this study, the plasma neutralisation analysis and have contributed to revisions of the work. **MKS and SF** supported the analysis and interpretation of data underlying this study as well as drafting and revisions. **NM, JN and CK** supported acquisition of clinical samples for this work and supported drafting and revisions of the manuscript. **AART, SLRJ, LH, OMK, DGW, SJD, TIdS and AH** made substantial contributions to conception/design and implementation of this work and/or acquisition of clinical samples for this work. They have supported drafting and revisions of the manuscript. **EH and JKQ** made substantial contributions to study design as well as data access, linkage and analysis. They have had direct access to and have verified clinical data used in this study. They have also supported drafting and revisions of this work. **ABD** made substantial contributions to study design as well as data access, linkage and analysis. They have also supported drafting and revisions of this work **JDC, LPH, AH, BR, KP and MM** made substantial contributions to conception and design of this work and have supported drafting and revisions of this work. **RAE and LVW** are co-leads of PHOSP-COVID, made substantial contributions to conception and design of this work and have supported drafting and revisions of this work. **JKB** obtained funding, is ISARIC4C consortium co–lead, has made substantial contributions to conception and design of this work and has supported drafting and revisions of this work. **MGS** obtained funding, is ISARIC4C consortium co–lead, sponsor/protocol chief investigator, has made substantial contributions to conception and design of this work and has supported drafting and revisions of this work. **CB** is the chief investigator of PHOSP-COVID and has made substantial contributions to conception and design of this work. **RST** and **LT** made substantial contributions to acquisition, analysis and interpretation of the data underlying this study and have contributed to drafting and revisions of this work. **LT** was the lead investigator of the PHOSP immunology sub-study. **PJMO** obtained funding, is ISARIC4C consortium co–lead, sponsor/protocol chief investigator, and has made substantial contributions to conception and design of this work. They have also made key contributions to interpretation of data and have co-written this manuscript. **FL and RST** have verified the data underlying this study. All authors have had full access to data and have read and approve the final version to be published. All authors agree to accountability for all aspects of this work.

All investigators within ISARIC4C and the PHOSP-COVID consortia have made substantial contributions to the conception or design of this study and/or acquisition of data for this study. The full list of authors within these groups is available in the supplementary materials.

## Data sharing statement

This is an Open Access article under the CC BY 4.0 license

The ISARIC4C protocol, data sharing and publication policy are available at https://isaric4c.net. ISARIC4C's Independent Data and Material Access Committee welcomes applications for access to data and materials (https://isaric4c.net).

The PHOSP-COVID protocol, consent form, definition and derivation of clinical characteristics and outcomes, training materials, regulatory documents, information about requests for data access, and other relevant study materials are available online: https://phosp.org/resource/. Access to these materials can be granted by contacting phosp@leicester.ac.uk and Phospcontracts@leicester.ac.uk.

All data used in this study is available within ODAP and accessible under reasonable request. Data access criteria and information about how to request access is available online: https://phosp.org/resource/. If criteria are met and a request is made, access can be gained by signing the eDRIS user agreement.

## Declaration of interests

FL, ST, AC, BJW, SS, NL, MKS, DS, JKS, CE, SCM, CD, CK, NM, JN, EH, ABD, JKQ, LPH, KP, LH, OMK, SF, TIdS, DGW, RST and JKB have no conflicts of interest. AART receives speaker fees and support to attend meetings from Janssen Pharmaceuticals. SLRJ is on the data safety monitoring board for Bexero trial in HIV + adults in Kenya. JDC is the deputy chief editor of ERS and receives consulting fees from AstraZeneca, Boehringer Ingelheim, Chiesi, GlaxoSmithKline, Insmed, Janssen, Novartis, Pfizer and Zambon. AH is Deputy chair of NIHR Translational Research Collaboration (unpaid role). BR receives honoraria from Axcella therapeutics. SJD is a member of the PITCH Consortium which has received funding from UK Department of Health and Social Care. SJD receives support from UKRI as part of “Investigation of proven vaccine breakthrough by SARS-CoV-2 variants in established UK healthcare worker cohorts: SIREN consortium & PITCH Plus Pathway” (MR/W02067X/1), with contributions from UKRI/NIHR through the UK Coronavirus Immunology Consortium (UK–CIC), the Huo Family Foundation and The National Institute for Health & Care Research (UKRIDHSC COVID-19 Rapid Response Rolling Call, Grant Reference Number COV19-RECPLAS). S.J.D. is a Scientific Advisor to the Scottish Parliament on COVID-19 for which she receives a fee. RAE is co–lead of PHOSP-COVID and receives fees from Astrazenaca/Evidera for consultancy on Long Covid and from Astrazenaca for consultancy on digital health. RAE has received speaker fees from Boehringer in June 2021 and has held a role as European Respiratory Society Assembly 01.02 Pulmonary Rehabilitation secretary. RAE is on the American Thoracic Society Pulmonary Rehabilitation Assembly programme committee. LVW receives funding from Orion pharma and GSK and holds contracts with Genentech and AstraZenaca. LVW has received consulting fees from Galapagos and Boehringer, is on the data advisory board for Galapagos and is Associate Editor for European Respiratory Journal. AH is a member of NIHR Urgent Public Health Group (June 2020–March 2021). MM is an applicant on the PHOSP study funded by NIHR/DHSC. MGS acts as an independent external and non-remunerated member of Pfizer's External Data Monitoring Committee for their mRNA vaccine program(s), is Chair of Infectious Disease Scientific Advisory Board of Integrum Scientific LLC, Greensboro, NC, USA and is director of MedEx Solutions Ltd and majority owner of MedEx Solutions Ltd and minority owner of Integrum Scientific LLC, Greensboro, NC, USA. MGS's institution has been gifted Clinical Trial Investigational Medicinal Product from Chiesi Farmaceutici S.p.A. MGS is a non-renumerated member of HMG UK New Emerging Respiratory Virus Threats Advisory Group (NERVTAG) and has previously been a non-renumerated member of SAGE. CB has received consulting fees and/or grants from GSK, AZ, Genentech, Roche, Novartis, Sanofi, Regeneron, Chiesi, Mologic and 4DPharma. LT has received consulting fees from MHRA and speak fees from Eisai Ltd. LT has a patent pending with ZikaVac. PJMO reports grants from the EU Innovative Medicines Initiative (IMI) 2 Joint Undertaking during the submitted work; grants from UK Medical Research Council, GlaxoSmithKline, Wellcome Trust, EU-IMI, UK, National Institute for Health Research, and UK Research and Innovation-Department for Business, Energy and Industrial Strategy; and personal fees from Pfizer, Janssen, and Seqirus, outside the submitted work.

## Appendix A Supplementary data

Supplemental Figs. S1–S9

Captions for supplementary materials

ISARIC 4C author_list

PHOSP_Collaborative_Group_Authors_List
