# Supplementary material for: SARS-CoV-2-specific nasal IgA wanes 9 months after hospitalisation with COVID-19 and is not induced by subsequent vaccination
Source: eBioMedicine. 2022 Dec 19;87:104402. doi: 10.1016/j.ebiom.2022.104402 (PMC9762734; doi:10.1016/j.ebiom.2022.104402)
Supplement: Supplemental Figs. S1–S9 [file mmc1.pdf]

Figure S1.

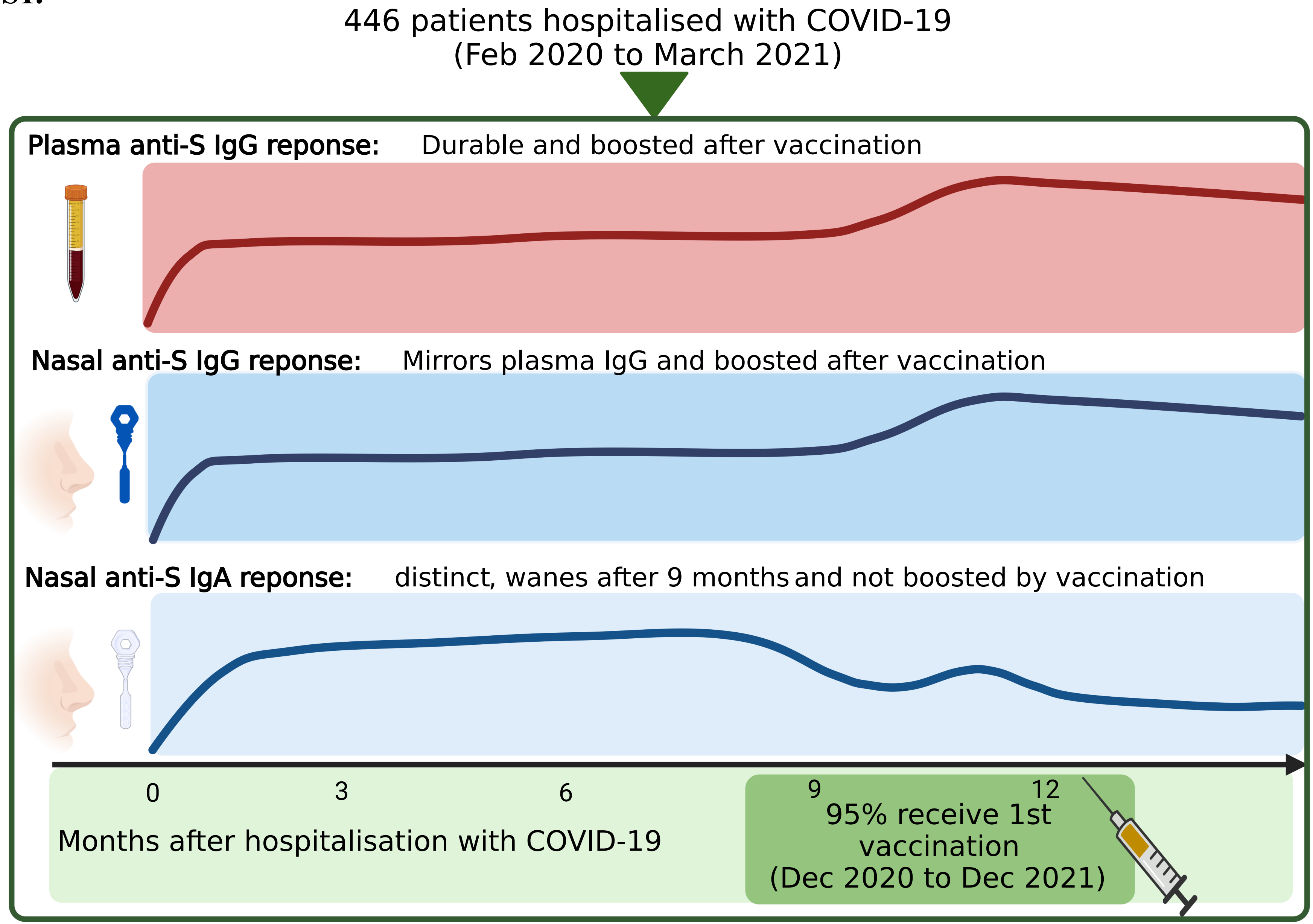
446 patients hospitalised with COVID-19
(Feb 2020 to March 2021)
Plasma anti-S IgG reponse: Durable and boosted after vaccination
Nasal anti-S IgG reponse: Mirrors plasma IgG and boosted after vaccination
Nasal anti-S IgA reponse: distinct, wanes after 9 months and not boosted by vaccination
0
3
6
9
12
Months after hospitalisation with COVID-19
95% receive 1st vaccination
(Dec 2020 to Dec 2021)

Figure S2.

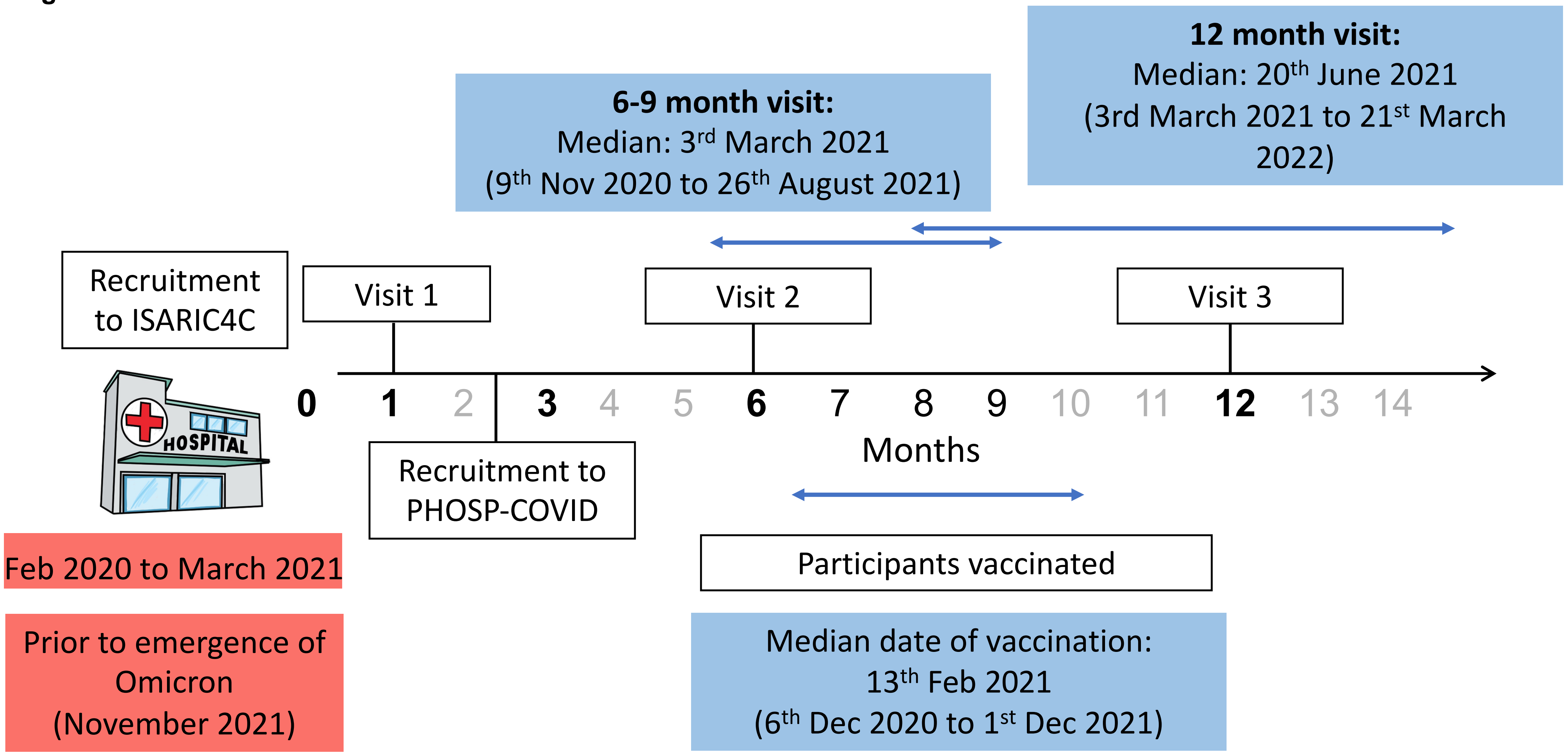

6-9 month visit:
Median: 3rd March 2021
(9th Nov 2020 to 26th August 2021)
12 month visit:
Median: 20th June 2021
(3rd March 2021 to 21st March 2022)
Recruitment to ISARIC4C
HOSPITAL
Visit 1
Visit 2
Visit 3
0 1 2 3 4 5 6 7 8 9 10 11 12 13 14
Months
Recruitment to PHOSP-COVID
Feb 2020 to March 2021
Prior to emergence of Omicron (November 2021)
Participants vaccinated
Median date of vaccination:
13th Feb 2021
(6th Dec 2020 to 1st Dec 2021)

Figure S3.

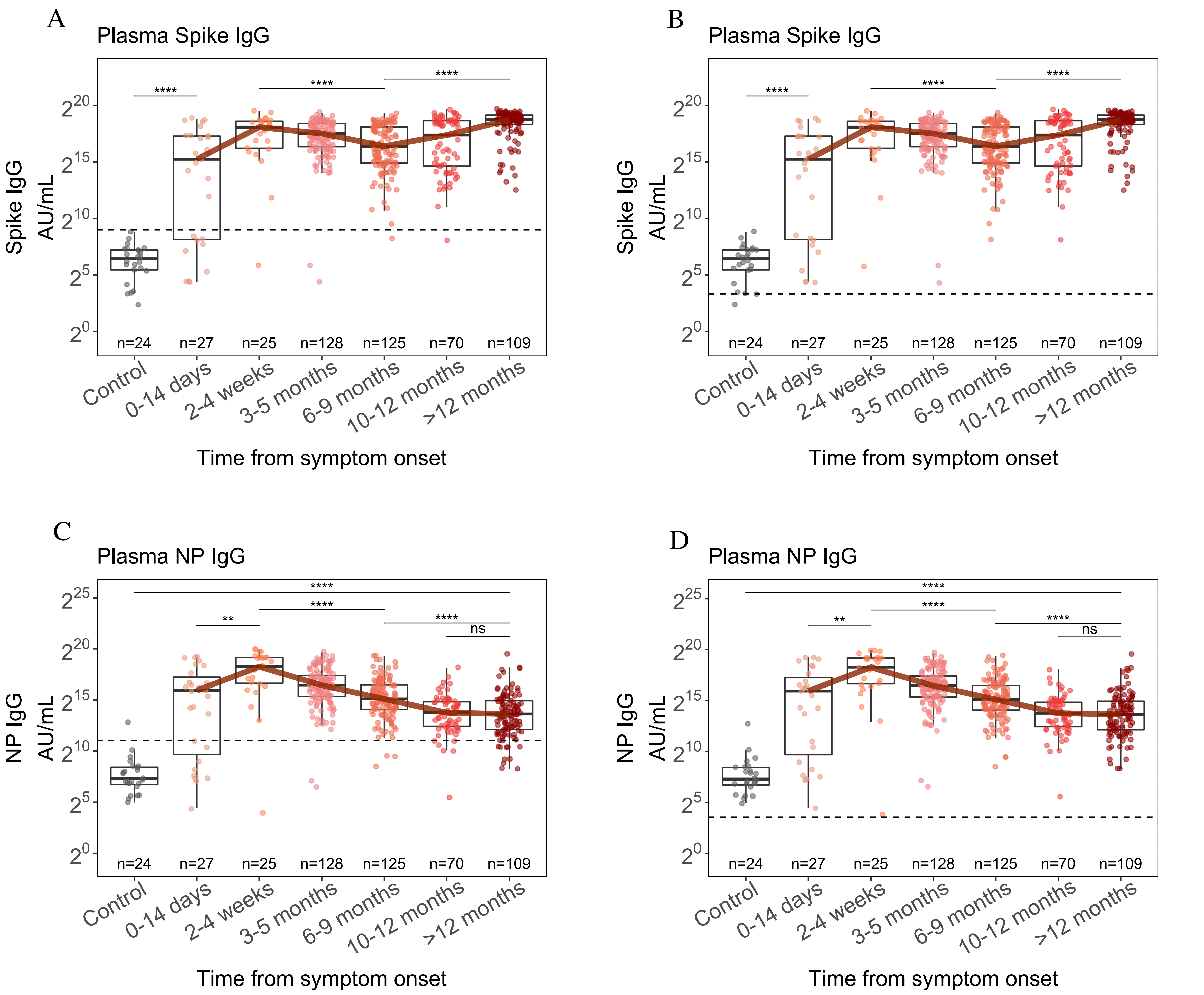
A
Plasma Spike IgG
Spike IgG AU/mL
****
n=24
n=27
n=25
n=128
n=125
n=70
n=109
Control
0-14 days
2-4 weeks
3-5 months
6-9 months
10-12 months
>12 months
Time from symptom onset
B
Plasma Spike IgG
Spike IgG AU/mL
C
Plasma NP IgG
NP IgG AU/mL
**
ns
D
Plasma NP IgG
NP IgG AU/mL

Figure S4.

A

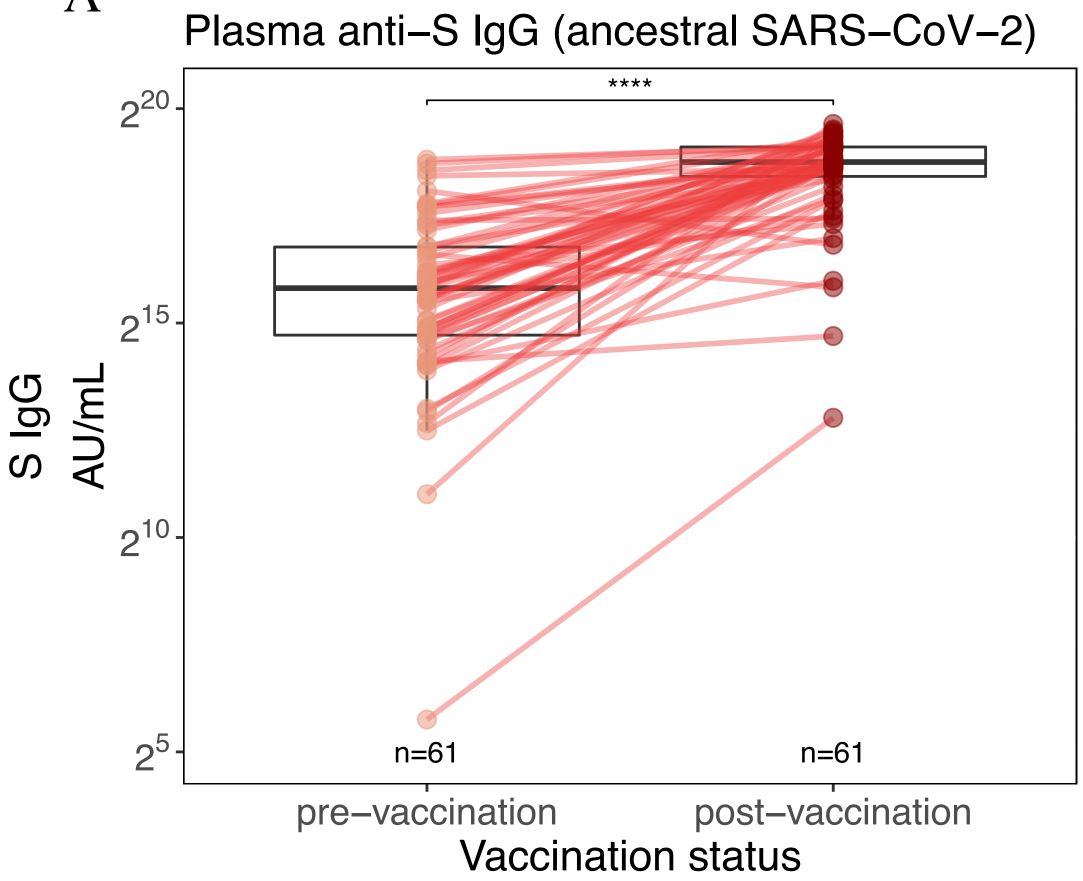

B

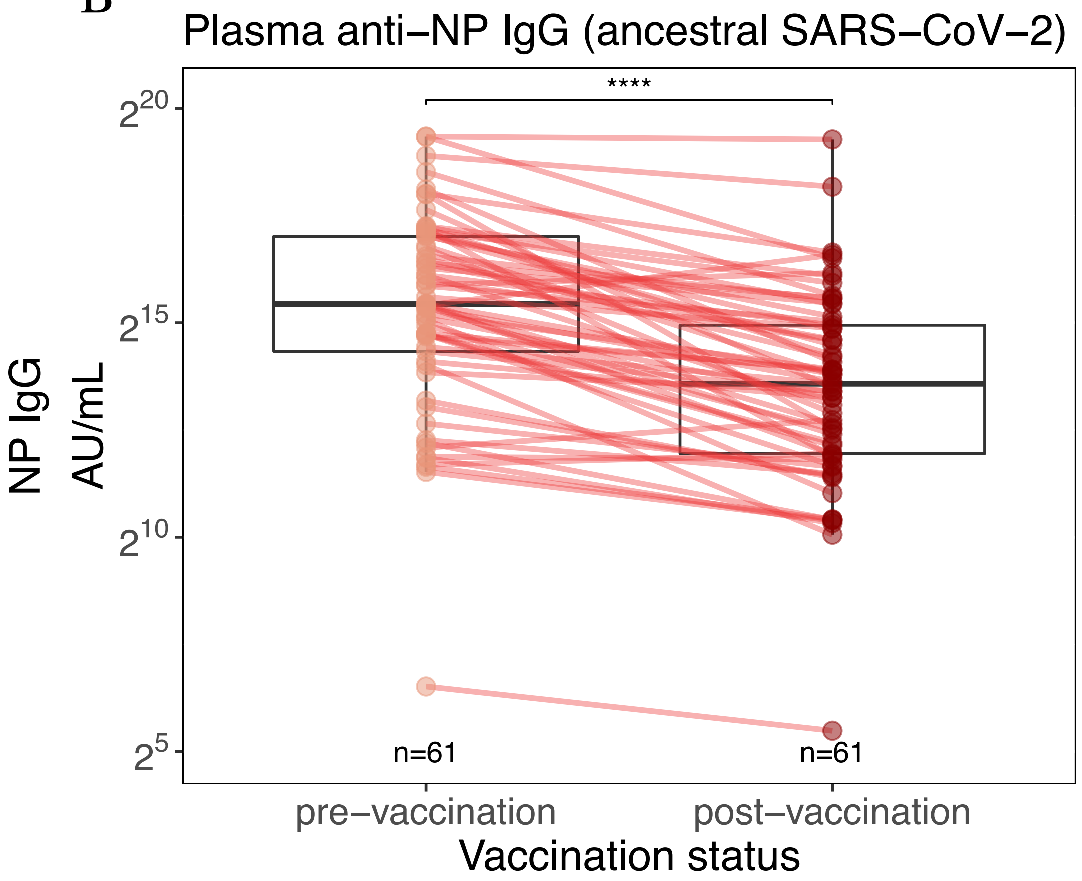

C

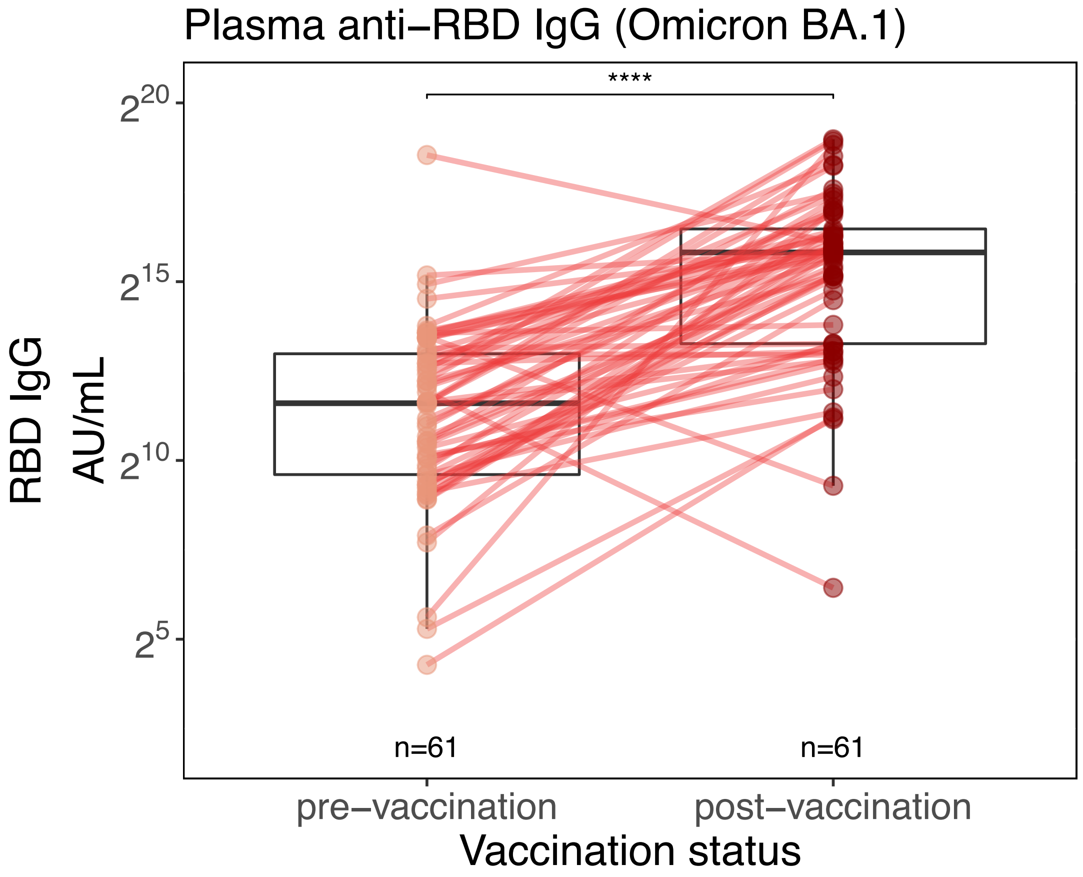

D

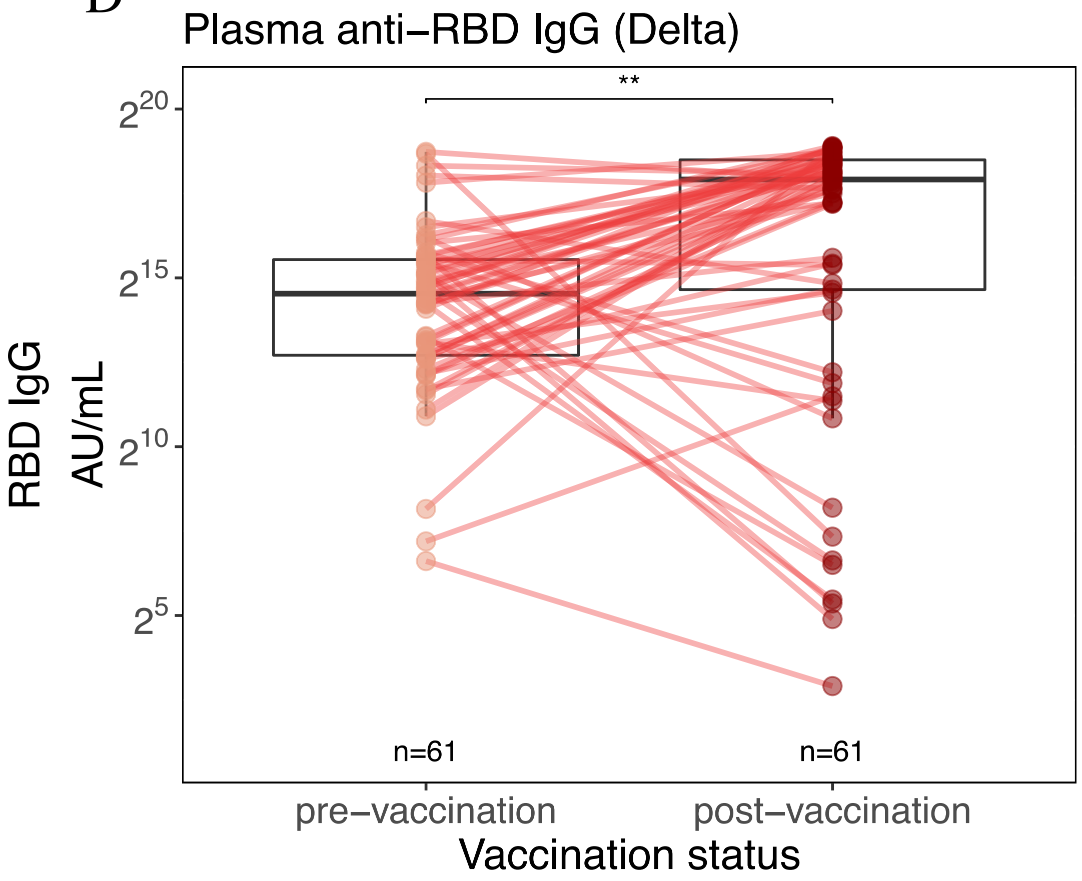

Figure S5.

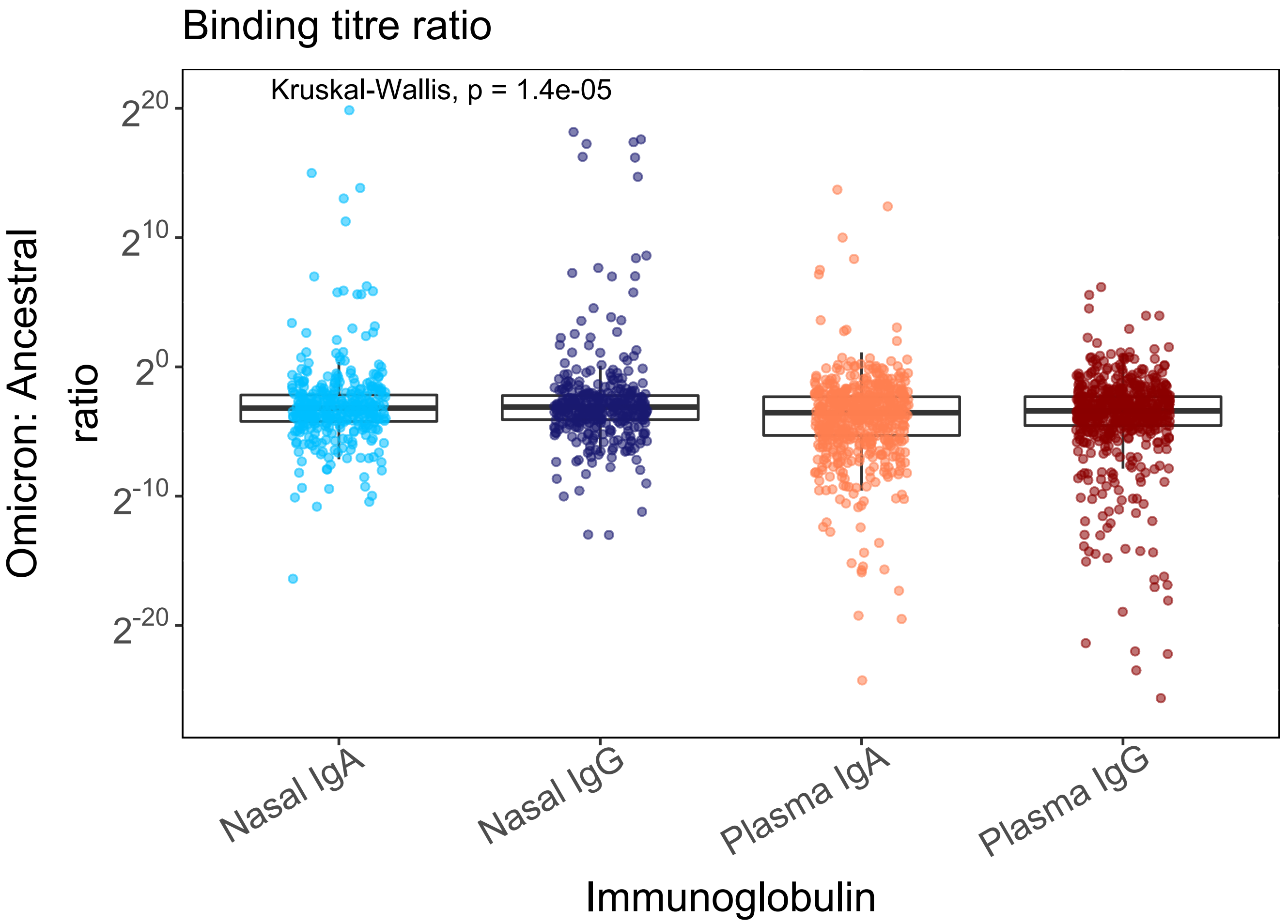

Binding titre ratio
Kruskal-Wallis, p = 1.4e-05
2^20
2^10
2^0
2^-10
2^-20
Omicron: Ancestral ratio
Nasal IgA
Nasal IgG
Plasma IgA
Plasma IgG
Immunoglobulin

Figure S6.

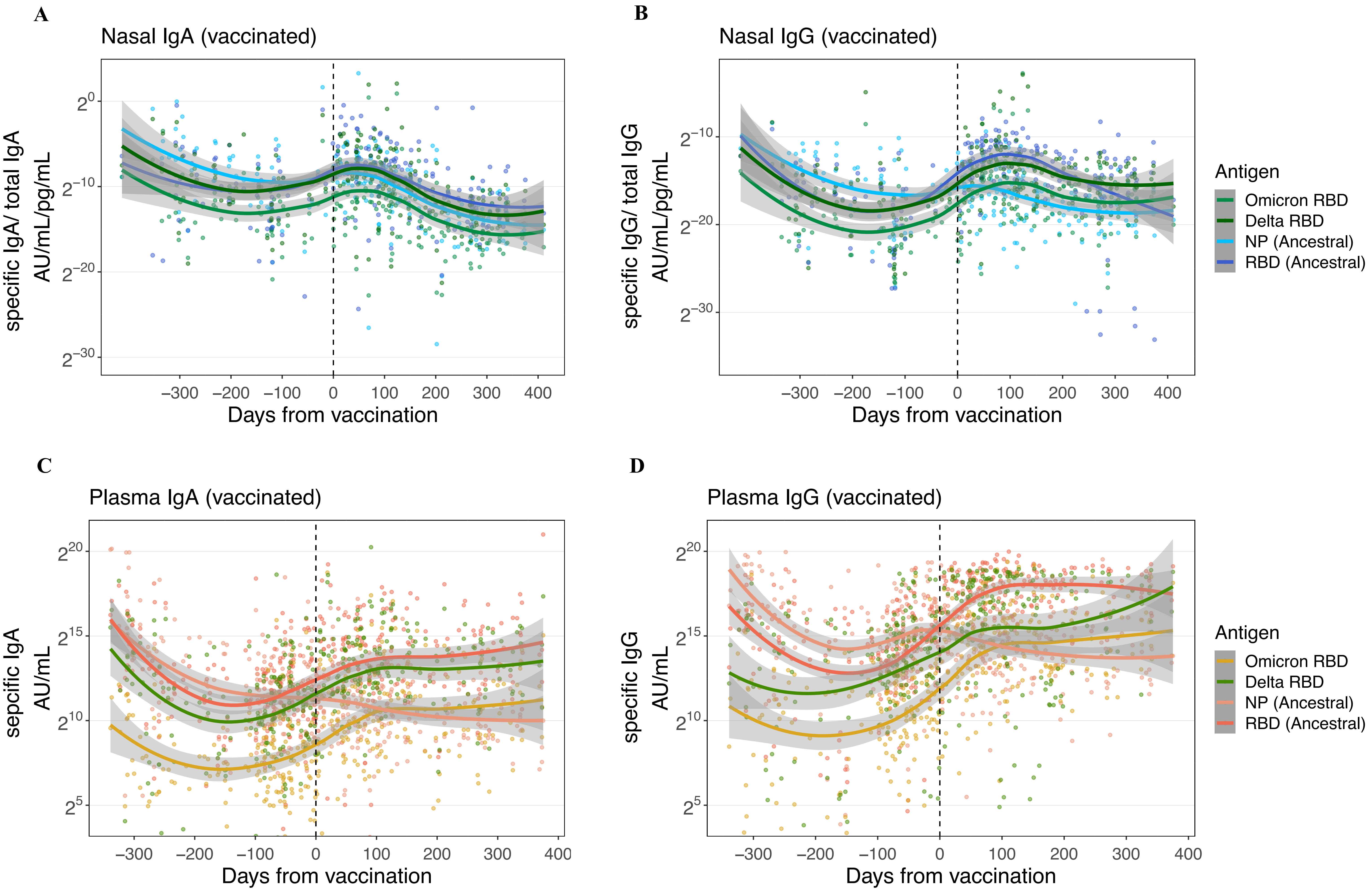
A
Nasal IgA (vaccinated)
specific IgA/ total IgA AU/mL/pg/mL
2^{0}
2^{-10}
2^{-20}
2^{-30}
−300 −200 −100 0 100 200 300 400
Days from vaccination
B
Nasal IgG (vaccinated)
specific IgG/ total IgG AU/mL/pg/mL
2^{-10}
2^{-20}
2^{-30}
−300 −200 −100 0 100 200 300 400
Days from vaccination
Antigen
Omicron RBD
Delta RBD
NP (Ancestral)
RBD (Ancestral)
C
Plasma IgA (vaccinated)
sepcific IgA AU/mL
2^{20}
2^{15}
2^{10}
2^{5}
−300 −200 −100 0 100 200 300 400
Days from vaccination
D
Plasma IgG (vaccinated)
specific IgG AU/mL
2^{20}
2^{15}
2^{10}
2^{5}
−300 −200 −100 0 100 200 300 400
Days from vaccination
Antigen
Omicron RBD
Delta RBD
NP (Ancestral)
RBD (Ancestral)

Figure S7.

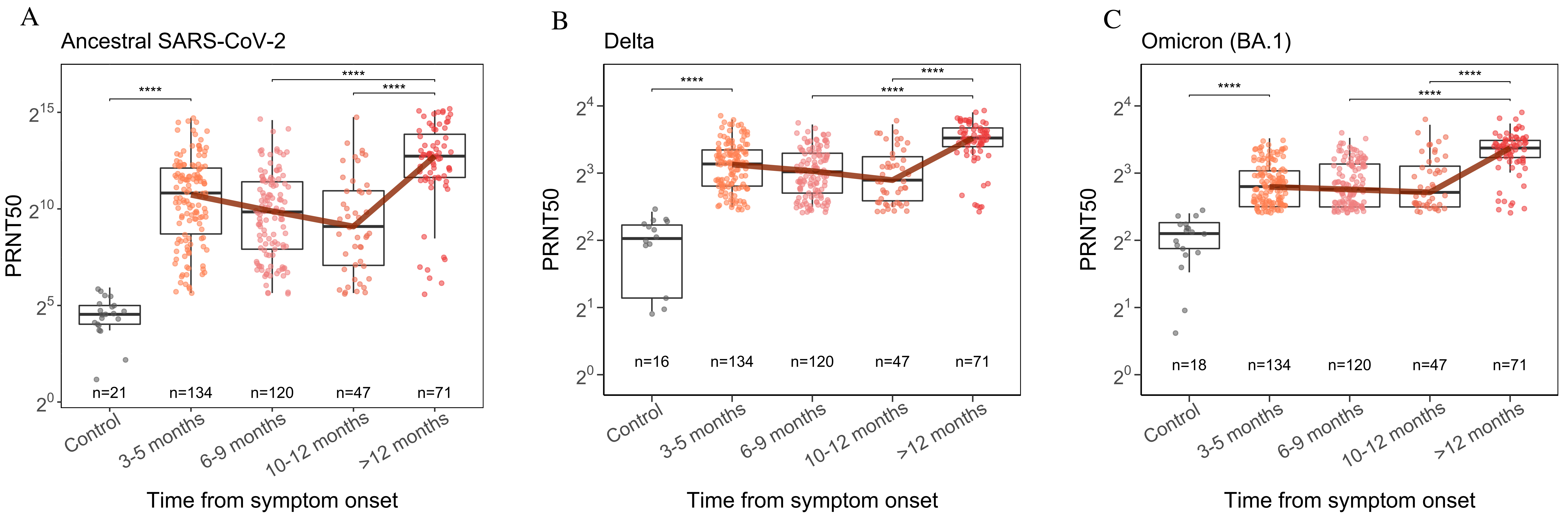
A
Ancestral SARS-CoV-2
PRNT50
2^15
2^10
2^5
2^0
****
****
****
n=21
n=134
n=120
n=47
n=71
Control
3-5 months
6-9 months
10-12 months
>12 months
Time from symptom onset
B
Delta
PRNT50
2^4
2^3
2^2
2^1
2^0
****
****
****
n=16
n=134
n=120
n=47
n=71
Control
3-5 months
6-9 months
10-12 months
>12 months
Time from symptom onset
C
Omicron (BA.1)
PRNT50
2^4
2^3
2^2
2^1
2^0
****
****
****
n=18
n=134
n=120
n=47
n=71
Control
3-5 months
6-9 months
10-12 months
>12 months
Time from symptom onset

Figure S8.

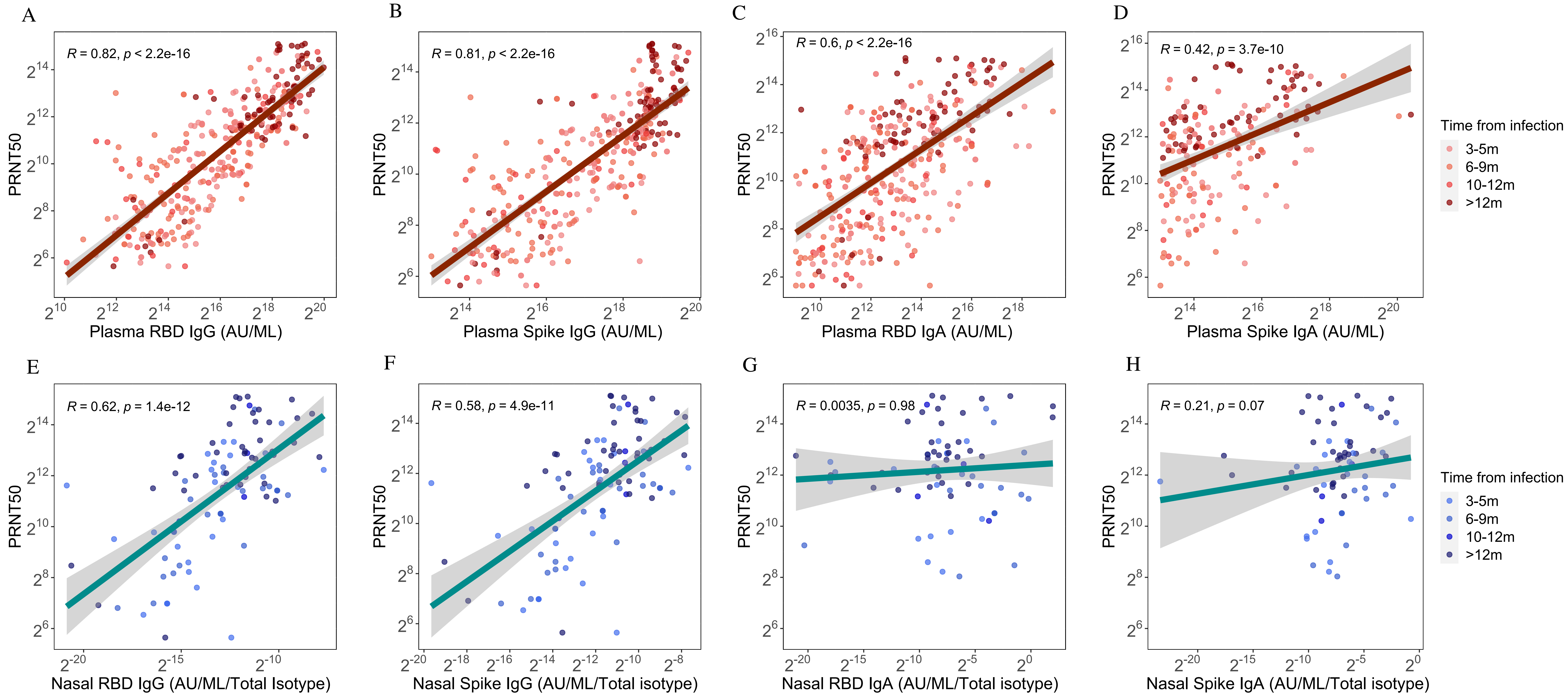
A
R = 0.82, p < 2.2e-16
PRNT50
Plasma RBD IgG (AU/ML)
B
R = 0.81, p < 2.2e-16
PRNT50
Plasma Spike IgG (AU/ML)
C
R = 0.6, p < 2.2e-16
PRNT50
Plasma RBD IgA (AU/ML)
D
R = 0.42, p = 3.7e-10
PRNT50
Plasma Spike IgA (AU/ML)
Time from infection
3-5m
6-9m
10-12m
>12m
E
R = 0.62, p = 1.4e-12
PRNT50
Nasal RBD IgG (AU/ML/Total Isotype)
F
R = 0.58, p = 4.9e-11
PRNT50
Nasal Spike IgG (AU/ML/Total isotype)
G
R = 0.0035, p = 0.98
PRNT50
Nasal RBD IgA (AU/ML/Total isotype)
H
R = 0.21, p = 0.07
PRNT50
Nasal Spike IgA (AU/ML/Total isotype)
Time from infection
3-5m
6-9m
10-12m
>12m

Figure S9.

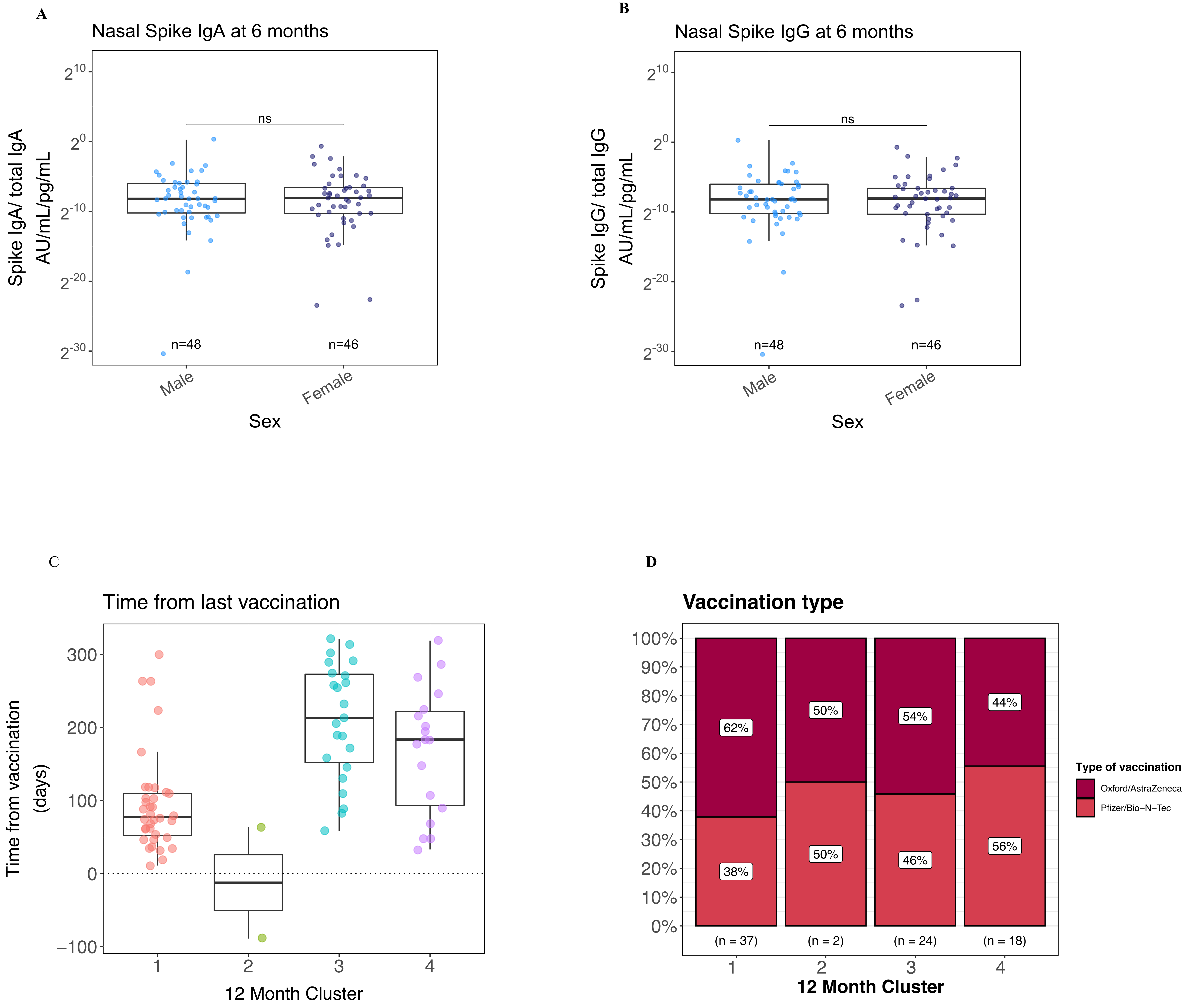
A
Nasal Spike IgA at 6 months
Spike IgA/ total IgA AU/mL/pg/mL
ns
n=48
n=46
Male
Female
Sex
B
Nasal Spike IgG at 6 months
Spike IgG/ total IgG AU/mL/pg/mL
ns
n=48
n=46
Male
Female
Sex
C
Time from last vaccination
Time from vaccination (days)
12 Month Cluster
D
Vaccination type
62%
38%
50%
50%
54%
46%
44%
56%
(n = 37)
(n = 2)
(n = 24)
(n = 18)
12 Month Cluster
Type of vaccination
Oxford/AstraZeneca
Pfizer/Bio-N-Tec
