# Supplementary material for: SARS-CoV-2-specific nasal IgA wanes 9 months after hospitalisation with COVID-19 and is not induced by subsequent vaccination
Source: eBioMedicine. 2022 Dec 19;87:104402. doi: 10.1016/j.ebiom.2022.104402 (PMC9762734; doi:10.1016/j.ebiom.2022.104402)
Supplement: Captions for supplementary materials [file mmc2.docx]

**Captions for supplementary figures**

**Figures S1. Graphical abstract.** Plasma and nasal samples collected at serial intervals from 446 adults hospitalised for COVID-19. Plasma and nasal IgG responses are durable and boosted by vaccination. Nasal IgA responses are compartmentalised from plasma IgG responses and are minimally affected by vaccination. This image was created with BioRender.com

**Figure S2. Schematic of study design.** Clinical data, plasma and/or nasal samples were obtained during hospital admission and/or 1 to 3 visits during convalescence. The 6 to 9 month visit coincided with the start of the UK vaccination campaign. Vaccination dates are shown as median (range) for individuals where vaccination status was known. Dates in which all study participants attended their 6 to 9 month and >12 month visit are shown in median (range).

**Figure S3.** **Comparison of thresholds for positivity**. A) Plasma anti-S IgG titres relative to threshold defined by mean+2SD of pre-pandemic samples (horizontal dotted line) and compared to the WHO threshold titre for seropositivity (horizontal dotted line) (B). The same comparison is shown for anti-NP plasma IgG responses (C­–D).

**Figure S4.** **Plasma antibody responses before and after vaccination.** Paired plasma IgG responses to S (A), NP (B) and RBD of Omicron BA.1 (C) and Delta (D) variant, taken before and after vaccination. * = p<0·05, ** = p<0·01, *** = p<0·001, **** = p<0·0001.

**Figure S5.** **Cross-reactivity of nasal and plasma antibody with Omicron virus after non-Omicron infection.** Ratio of binding titre to RBD of Omicron BA.1 variant and ancestral SARS-CoV-2 across nasal and plasma compartments.

**Figure S6.** **Antibody responses to variants of concern in individuals infected with non-Omicron virus and subsequently vaccinated.** Trajectory of Nasal IgA (A), nasal IgG (B), plasma IgA (C) and plasma IgG (D) responses to RBD of Omicron (BA.1) and Delta variant before and after vaccination. Responses to NP and RBD of ancestral SARS-CoV-2 are also shown for comparison. Trajectories have been modelled using a LOESS regression curve and 95% confidence intervals are shown in grey. The vertical dashed line indicates the time of vaccination.

**Figure S7.** **Plasma neutralising titres between 3 and 12 months after infection.** Neutralisation of ancestral SARS-CoV-2 (A), Delta variant (B) and Omicron variant (C) are shown. The red line indicates the trajectory of the median titre across each time bin. * = p<0·05, ** = p<0·01, *** = p<0·001, **** = p<0·0001.

**Figure S8.** **The relationship between plasma neutralising titres and nasal and plasma binding antibody.** Correlation between plasma neutralising titre and plasma IgG (A–B) and IgA (C–D) binding titre to RBD and S are shown. The correlation between plasma neutralising titre and nasal IgG (E–F) and IgA (G–H) binding titre to RBD and S are also shown. A regression line has been fit to the data for which the 95% confidence intervals are shown in grey. *R*=Spearman-rank correlation coefficient. PRNT_50_ = serum dilution resulting in >50% reduction in infectivity.

**Figure S9.** **Clinical variables associated with nasal and plasma antibody titre at 6 months.** The nasal IgA (A) and IgG (B) response divided by sex. Panel C) depicts time from vaccination in clusters 1-4 derived from unsupervised, hierarchical clustering analysis of nasal IgA, plasma IgA and plasma IgG anti-S and anti-RBD responses at 6 months from symptom onset. Panel B) shows the proportion of individuals vaccinated with either Pfizer/Bio-N-Tec (BNT162b2) or Oxford/ AstraZeneca (ChAdOx1 nCoV-19) vaccine in each cluster.
